# The prevalence of mental ill-health in women during pregnancy and after childbirth during the Covid-19 pandemic: a systematic review and Meta-analysis

**DOI:** 10.1186/s12884-022-05243-4

**Published:** 2023-01-28

**Authors:** Gayathri Delanerolle, Mary McCauley, Martin Hirsch, Yutian Zeng, Xu Cong, Heitor Cavalini, Sana Sajid, Ashish Shetty, Shanaya Rathod, Jian Qing Shi, Dharani K. Hapangama, Peter Phiri

**Affiliations:** 1 Nuffield Department of Primary Health Care Sciences, Uuniversity of Oxford, Oxford, UK; 2 Southern Health NHS Foundation Trust, Research and Innovation Department, Clinical Trials Facility, Tom Rudd Unit Moorgreen Hospital, Botley Road, West End, Southampton, SO30 3JB UK; 3 Liverpool Women’s NHS Foundation Trust, Liverpool, UK; 4 University College London, London, UK; 5 Oxford University Hospitals NHS Foundation Trust, Oxford, UK; 6 Southern University of Science and Technology, Shenzhen, China; 7 University College London Hospitals NHS Foundation Trust, London, UK; 8 National Center for Applied Mathematics, Shenzhen, China; 9 University of Liverpool, Liverpool, UK; 10 School of Psychology, Faculty of Environmental and Life Sciences, University of Southampton, Southampton, UK

**Keywords:** Covid-19, Mental ill-health, Depression, Anxiety, Stress, Pregnancy, Antenatal care, Wellbeing

## Abstract

**Background:**

This systematic review aims to explore the prevalence of the impact of the COVID-19, MERS, and SARS pandemics on the mental health of pregnant women.

**Methods:**

All COVID-19, SARS and MERS studies that evaluated the mental health of pregnant women with/without gynaecological conditions that were reported in English between December 2000 – July 2021 were included. The search criteria were developed based upon the research question using PubMed, Science Direct, Ovid PsycINFO and EMBASE databases. A wide search criterion was used to ensure the inclusion of all pregnant women with existing gynaecological conditions. The Newcastle-Ottawa-Scale was used to assess the risk of bias for all included studies. Random effects model with restricted maximum-likelihood estimation method was applied for the meta-analysis and I-square statistic was used to evaluate heterogeneity across studies. The pooled prevalence rates of symptoms of anxiety, depression, PTSD, stress, and sleep disorders with 95% confidence interval (CI) were computed.

**Results:**

This systematic review identified 217 studies which included 638,889 pregnant women or women who had just given birth. There were no studies reporting the mental health impact due to MERS and SARS. Results showed that women who were pregnant or had just given birth displayed various symptoms of poor mental health including those relating to depression (24.9%), anxiety (32.8%), stress (29.44%), Post Traumatic Stress Disorder (PTSD) (27.93%), and sleep disorders (24.38%) during the COVID-19 pandemic.

**Discussion:**

It is important to note that studies included in this review used a range of outcome measures which does not allow for direct comparisons between findings. Most studies reported self-reported measure of symptoms without clinical diagnoses so conclusions can be made for symptom prevalence rather than of mental illness. The importance of managing mental health during pregnancy and after-delivery improves the quality of life and wellbeing of mothers hence developing an evidence-based approached as part of pandemic preparedness would improve mental health during challenging times.

**Other:**

The work presented in this manuscript was not funded by any specific grants**.** A study protocol was developed and published in PROSPERO (CRD42021235356) to explore several key objectives.

## Background

In December 2019, SARS-CoV-2 unprecedentedly spread around the world, overwhelming global healthcare systems. On March 11, 2020, the World Health Organization declared the coronavirus disease 2019 (COVID-19) global pandemic. This led to a rippling impact of the virus on healthcare systems. In order to reduce viral transmission and relieve pressure on healthcare networks, many countries, including the United Kingdom (UK), entered a national lockdown by which people were ordered by law to stay at home [1. ]. In many hospitals, staff were redeployed, and departments were adapted or converted to provide COVID-19 services [1. ].

Whilst public health emergencies explicitly effect the physical health of a population, increased levels of poor mental health can also be discovered (e.g., depression, PTSD, substance use disorder, and behavioural disorders) [2. ]. Influences directly related to infection, such as, the neuroinvasive potential of SARS-CoV-2, may affect brain function and in turn mental health. The treatment for COVID-19 may also have adverse effects on mental health. For example, the imposition of unfamiliar public health measures (i.e., social isolation) increases the likelihood of clinically significant depression or anxiety [2. , 3. ]. Whilst all individuals were urged to comply with lockdown protocols, emotional distress tempted some to consider violating the recommended public health measures [3. ].

One vulnerable group during the pandemic were pregnant women and women who had recently given birth. Millions of women experience mental ill-health during pregnancy and after childbirth, with maternal mental ill-health being an international public health concern, affecting up to 10% of women during pregnancy and 13% of women after childbirth [4. –6. ]. Compromised mental health can cause short and long-term consequences for the mother and baby however limited data exists on the prevalence of mental ill-health in women who were pregnant and gave birth during the COVID-19 pandemic [6. –8. ].

This systematic review and meta-analysis will assess the prevalence of mental ill-health in women during pregnancy and after childbirth throughout the Covid-19 pandemic. Findings with be compared to other global pandemics including SARS and MERS.

## Methods

A systematic methodology was developed along with a relevant protocol that was peer reviewed and published in PROSPERO (CRD42021235356).

### Search criteria

The search criteria were developed based upon the research question using PubMed, Science Direct, Ovid PsycINFO and EMBASE databases. A wide search criterion was used to ensure the inclusion of all pregnant women with existing gynaecological conditions. The MeSH terms used include (COVID) OR (SARS-CoV-2) AND (SARS) AND (MERS) AND ((mental health) OR (depression) OR (anxiety) OR (PTSD) OR (psychosis) OR (unipolar) OR (bipolar)) AND ((PCOS) OR (fibroid) OR (endometriosis) OR (pre-eclampsia) OR (still birth) OR (GDM) OR (preterm birth) OR (women’s health) OR (pregnant women) OR (pregnancy)).

### Screening eligibility criteria

**Fig. 1:**
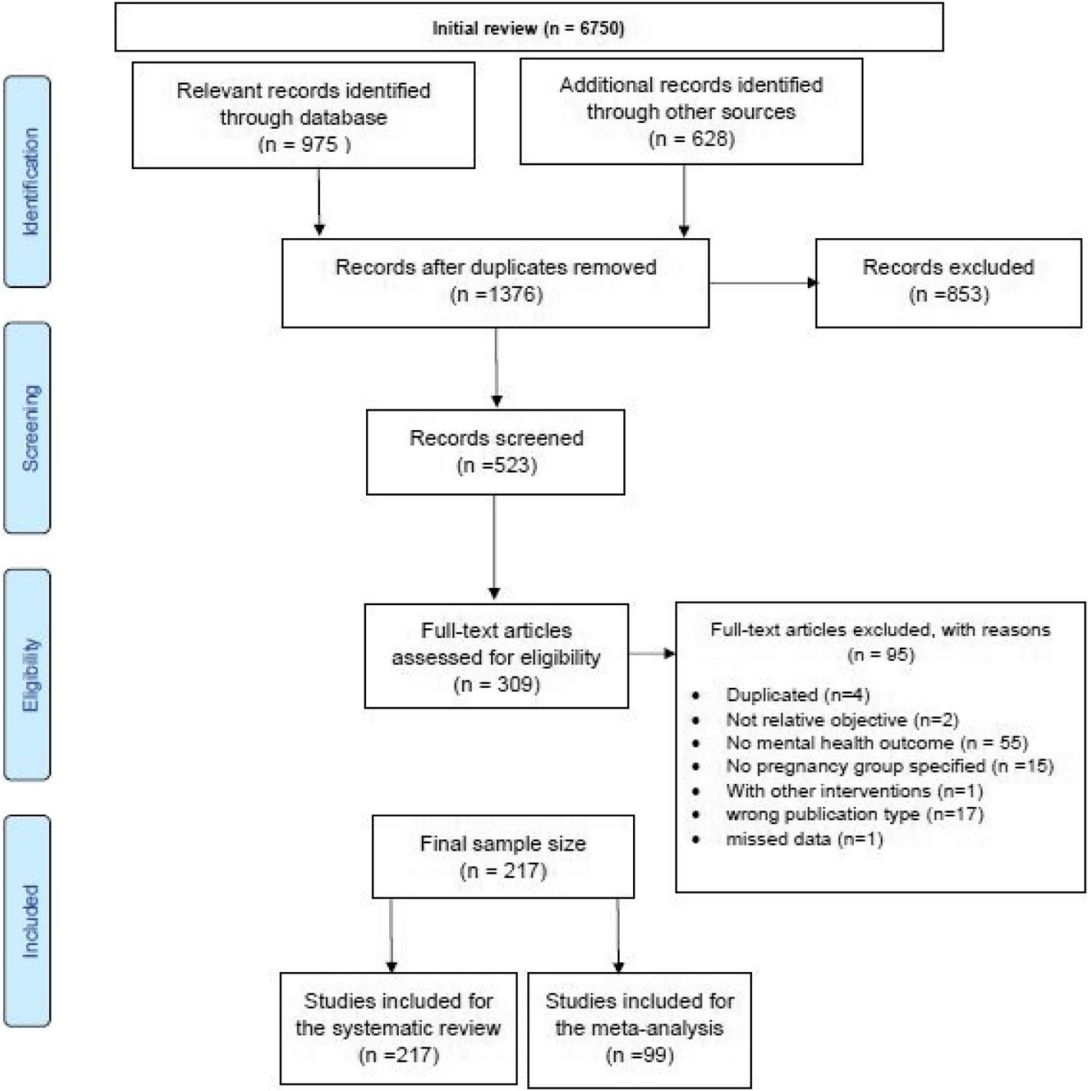
PRISMA Flow Chart outlining search strategy. Legend. The above PRISMA flow chart outlines the study search strategy for both the systematic review and meta-analysis

All studies published in English were included from 20th December 2019 to 31st July 2021. Screening and data extraction were performed by two authors independently. Initially, titles and abstracts were reviewed to determine the relevance. A PRISMA (Preferred Reporting Items for Systematic Reviews and Meta-analyses) diagram was completed based on the eligibility steps completed (see Fig. 1).

### Inclusion and exclusion criteria

All COVID-19, SARS and MERS studies that evaluated the mental health of pregnant women with/without gynaecological conditions that were reported in English between December 2000 – July 2021 were included. All other studies were excluded from this analysis.

### Data extraction

Full texts of the included papers were reviewed to extract the following data: time and locations of the study, participants demographics, sample size, mean age, gestation, days since childbirth, prevalence of mental symptoms, data collection tools used, and cut-offs scores applied. Any disagreements were discussed and resolved by consensus between two authors. For studies with both COVID-19 and non-COVID-19 cohort, we only used data of the COVID-19 cohort and the *p*-value comparing them. Studies from SARS and MERS were also reviewed in full to ensure the eligibility criteria was met. Studies reporting mean (SD) or median (IQR) of the scales measuring mental symptoms instead of prevalence rates were included and a simulation method assuming normal distribution was applied to generate the corresponding prevalence rates.

### Risk of bias (RoB) assessment

A risk of bias (RoB) table assessment was completed to demonstrate the risk of bias within the studies used in the systematic review and meta-analysis. The RoB is reflective of a fixed set of biases within domains of study design, conduct and reporting. This combined with the quality check allows the findings of the study to be scientifically justified, and clinically viable. The Newcastle-Ottawa-Scale (NOS) was used to assess the RoB for all systematically included studies as demonstrated within the RoB table (See Page 33, Table 1).

**Table 1:** Risk of Bias for all included studies

First author	Symptom(s)	Selection	Compara bility	Exposure /outcome	Total
Lebel C	Anxiety; depression	4	0	2	6
Ayaz R	Anxiety	3	0	3	6
Durankuş F	Anxiety; depression	3	0	2	5
Liu X	Anxiety	5	2	2	9
Mappa I	Anxiety	4	0	2	6
López-Morales H	Anxiety; depression	3	2	2	7
Salehi L	Anxiety	3	0	2	5
Gur RE	Anxiety; depression	4	2	2	8
Ng QJ	Anxiety; depression; stress	5	0	2	7
Effati-Daryani F	Anxiety; depression; stress	4	2	2	8
Ravaldi C	Anxiety	2	0	2	4
Zhou Y	Anxiety; depression; PTSD; sleep disorders	3	2	2	7
Kahyaoglu Sut H	Anxiety; depression	3	2	2	7
Sinaci S	Anxiety	4	1	2	7
Dong H	Anxiety; depression	3	2	2	7
Hocaoglu M	Anxiety; PTSD	4	0	3	7
Yue C	Anxiety	4	1	2	7
Taubman-Ben-Ari O	Anxiety	2	0	2	4
Maharlouei N	Anxiety	2	0	2	4
Milne SJ	Anxiety	1	0	1	2
Ceulemans M	Anxiety; depression; stress	4	2	2	8
Yassa M	Anxiety	3	0	1	4
Jiang H	Anxiety; depression; stress	4	2	2	8
Mayeur A	Anxiety	2	0	2	4
Lin W	Anxiety; depression	4	2	2	8
Yang X	Anxiety; depression	4	0	2	6
Akgor U	Anxiety; depression	4	0	2	6
Preis H	Anxiety; stress	3	2	2	7
Dagklis T	Anxiety; depression	4	0	2	6
Esteban-Gonzalo S	Anxiety	4	2	2	8
Koyucu RG	Anxiety; depression; stress	3	0	2	5
Liu J	Anxiety; depression	3	2	2	7
Cao Y	Anxiety; depression	3	0	2	5
Mappa I	Anxiety	4	0	2	6
Mehdizadehkashi A	Anxiety	4	0	3	7
Yirmiya K	Anxiety; depression; stress	3	2	2	7
Xie M	Anxiety; depression; sleep disorders	3	0	1	4
Ge Y	Anxiety	3	0	2	5
López-Morales H	Anxiety; depression	3	2	2	7
Puertas-Gonzalez JA	Anxiety; depression; stress	4	2	2	8
Çolak S	Anxiety; depression; stress	4	0	2	6
Xu K	Anxiety; depression; stress; sleep disorders	4	2	2	8
Zilver SJM	Anxiety; depression; stress	4	2	2	8
Maharlouei N	Anxiety; depression; stress	4	0	2	6
Harrison V	Anxiety; depression	4	0	2	6
Saadati N	Anxiety	4	0	2	6
Wang Q	Anxiety; depression	4	2	2	8
Behmard V	Anxiety	4	2	2	8
Hamzehgardeshi Z	Anxiety; depression	4	2	2	8
Jelly P	Anxiety	4	0	2	6
Wang Q	Anxiety; depression	5	0	2	7
Zhang Y	Anxiety; stress	4	1	2	7
Masjoudi M	Anxiety; stress	4	1	2	7
Shangguan F	Anxiety; stress	3	2	2	7
Tsakiridis I	Anxiety; depression	4	0	2	6
Brik M	Anxiety	1	0	1	2
Effati-Daryani F	Anxiety; depression; stress	4	0	2	6
Lubián López DM	Anxiety	4	0	2	6
Maleki A	Anxiety	4	0	2	6
Khoury JE	Anxiety; depression; stress; sleep disorders	4	2	2	8
Suárez-Rico BV	Anxiety	3	0	2	5
Obata S	Anxiety; depression	3	2	2	7
Mo PKH	Anxiety; depression	4	2	2	8
Wu F	Anxiety; depression	4	2	2	8
Ding W	Anxiety	4	0	2	6
Mirzaei N	Anxiety; depression	4	0	2	6
Ramirez Biermann C	Anxiety; depression	1	0	1	2
Palalioglu RM	Anxiety	3	0	2	5
Molgora S	Anxiety; depression	4	0	2	6
Patabendige M	Anxiety; depression	4	0	1	5
Zeng X	Anxiety; depression; sleep disorders	4	2	2	8
Nurrizka RH	Anxiety	2	0	2	4
Wu Y	Depression	4	2	3	9
Wang Y	Depression; PTSD	3	1	2	6
Medina-Jimenez V	Depression; stress	4	0	2	6
Matsushima M	Depression	4	0	2	6
Gildner TE	Depression	4	0	2	6
Shayganfard M	Depression; stress	4	0	2	6
Silverman ME	Depression	3	0	3	6
Muhaidat N	Depression	3	0	2	5
Thayer ZM	Depression	4	2	2	8
Zhang CJP	Depression; PTSD	4	2	2	8
Khamees RE	Depression	4	0	2	6
Silverman ME	Depression	3	0	2	5
Shahid A	Depression; sleep disorders	4	0	1	5
Ionio C	Depression	4	0	2	6
Overbeck G	Depression	3	2	2	7
Kachi Y	Depression	3	2	2	7
Smith CL	Depression; stress	2	2	2	6
King LS	Depression	2	2	1	5
Korukcu O	Depression	4	0	2	6
Zhou Y	Depression	4	2	2	8
Chaves C	Depression	4	0	2	6
Davis JA	Stress	4	2	3	9
Ionio C	PTSD	4	0	2	6
Basu A	PTSD	4	2	2	8
Kara P	PTSD	4	0	2	6
Wang J	Sleep disorders	4	2	2	8

The above table outlined the risk of bias results for all studies included within this paper. Firth author and symptoms of mental health are displayed. Four outcome measures assessing risk of bias are also shown

### Data analysis

Random effects model with restricted maximum-likelihood estimation method was applied for the meta-analysis and I-square statistic was used to evaluate heterogeneity across studies. The pooled prevalence rates of symptoms of anxiety, depression, PTSD, stress, and sleep disorders with 95% confidence interval (CI) were computed. Subgroup analysis was conducted based on pregnancy trimester. Sensitivity analysis was performed to assess the robustness of the results. Potential publication bias was assessed with funnel plot and Egger’s test. Analyses were conducted with the R studio (version 1.4.17.17) and STATA 16.1.

## Results

Our initial search identified a total of 1603 papers and 523 studies were excluded after screening by titles and abstracts. After full-text evaluation, 217 were included in the systematic review and 99 studies were included in the meta-analysis. The PRISMA flowchart was illustrated in Fig. 1.

### Characteristics of studies

A total of 217 COVID-19 studies were included and 99 studies were meta-analysed. These studies were reported from various parts of the world, as indicated in the characteristics (See page 35 for Tables 2 and 3). We did not identify SARS and MERS studies that were suitably aligned to the eligibility criteria of our study.

**Table 2:** Outline of all studies in the systematic review and meta-analysis

ID	Authors	Publication Year	Country	Sample size	***p***-value
1	Wu Y [9. ]	2020	China	1285	0.01
2	Durankuş F [10. ]	2020	Turkey	260	N/A
3	Moyer CA [11. ]	2020	United States	2740	*p* < 0.001
4	Zanardo V [12. ]	2020	Italy	91	*p* < 0.001
5	López-Morales H [13. ]	2021	Argentina	43	N/A
6	Salehi L [14. ]	2020	Iran	220	N/A
7	Pariente G [15. ]	2020	Israel	223	0.002
8	Ostacoli L [16. ]	2020	Italy	163	N/A
9	Ravaldi C [17. ]	2021	Italy	200	*p* < 0.001
10	Zhou Y [18. ]	2020	China	544	N/A
11	Kahyaoglu Sut H [19. ]	2021	Turkey	403	N/A
12	Hui PW [20. ]	2021	Hong Kong (China)	925	*p* < 0.05
13	Oskovi-Kaplan ZA [21. ]	2021	Turkey	223	N/A
14	Sinaci S [22. ]	2020	Turkey	246	N/A
15	Dong H [23. ]	2021	China	156	N/A
16	Hocaoglu M [24. ]	2020	Turkey	283	*p* = 0.01
17	Liang P [25. ]	2020	China	845	N/A
18	Preis H [26. ]	2020	US	4451	N/A
19	Yue C [27. ]	2021	China	308	N/A
20	Maharlouei N [28. ]	2020	Iran	540	N/A
21	Medina-Jimenez V [29. ]	2020	Mexico	503	N/A
22	Ceulemans M [30. ]	2020	Belgium	3445	N/A
23	Milne SJ [30. ]	2020	Ireland	70	N/A
24	Matsushima M [31. ]	2020	Japan	1777	N/A
25	Ceulemans M [32. ]	2021	Ireland, Norway, Switzerland, the Netherlands, and the UK	3545	N/A
26	Gildner TE [33. ]	2020	US	1856	N/A
27	Shayganfard M [34. ]	2020	Iran	103	N/A
28	Yassa M [35. ]	2020	Turkey	203	N/A
29	Silverman ME [36. ]	2020	US	516	*p* < 0.001
30	Muhaidat N [37. ]	2020	Jordan	944	N/A
31	Thayer ZM [38. ]	2021	US	2099	N/A
32	Jiang H [39. ]	2021	China	1873	N/A
33	Zhang Y [40. ]	2021	China	560	N/A
34	Mayeur A [41. ]	2020	France	88	N/A
35	Lin W [42. ]	2021	China	751	N/A
36	Zhang CJP [43. ]	2020	China	1901	N/A
37	Yang X [44. ]	2021	Chinese	19,515	N/A
38	Khamees RE [45. ]	2021	Egypt	120	*p* < 0.001
39	Lorentz MS [46. ]	2021	Brazil	50	*p* = 0.004 (comparing scores) *p* = 0.062 (comparing prevalence)
40	Silverman ME [47. ]	2020	US	485	N/A
41	Akgor U [48. ]	2021	Turkey	297	N/A
42	Shahid A [49. ]	2020	Pakistan	552	N/A
43	Preis H [50. ]	2020	US	788	N/A
44	Dagklis T [51. ]	2020	Greece	269	p < 0.001
45	Ionio C [52. ]	2021	Italy	40	N/A
46	Esteban-Gonzalo S [53. ]	2021	Spain	353	N/A
47	Koyucu RG [54. ]	2021	Turkey	729	N/A
48	Overbeck G [55. ]	2021	Denmark	330	0.2209
49	Kachi Y [56. ]	2021	Japan	270	N/A
50	Mariño-Narvaez C [57. ]	2021	Spain	75	*p* = 0.038
51	Liu J [58. ]	2021	US	715	N/A
52	Smith CL [59. ]	2021	USA	83	N/A
53	Cao Y [60. ]	2021	China	298	N/A
54	Mappa I [61. ]	2021	Italy	161	*p* < 0.0001
55	Mehdizadehkashi A [62. ]	2021	Iran	300	N/A
56	Yirmiya K [63. ]	2021	Israel	1114	N/A
57	Xie M [64. ]	2021	China	689	*p* = 0.03
58	Ge Y [65. ]	2021	China	446	N/A
59	López-Morales H [66. ]	2021	Argentina	102	N/A
60	Puertas-Gonzalez JA [67. ]	2021	Spain	100	*p* = 0.025
61	Çolak S [68. ]	2021	Turkey	149	N/A
62	Xu K [69. ]	2021	China	274	N/A
63	Zilver SJM [70. ]	2021	Netherlands	1102	*p* = 0.14(comparing prevalence)/*p* = 0.03(comparing score)
64	Maharlouei N [71. ]	2021	Iran	540	N/A
65	Harrison V [72. ]	2021	UK	205	N/A
66	Saadati N [73. ]	2021	Iran	300	N/A
67	Wang Q [74. ]	2021	China	15,428	N/A
68	Behmard V [75. ]	2021	Iran	801	N/A
69	King LS [76. ]	2021	US	725	*p* < 0.001
70	Nurrizka RH [77. ]	2021	Indonesia	120	N/A
71	Jelly P [78. ]	2021	India	333	N/A
72	Wang Q [79. ]	2021	China	19,515	N/A
73	Zhang Y [80. ]	2021	China	1794	N/A
74	Masjoudi M [81. ]	2021	Iran	215	N/A
75	Shangguan F [82. ]	2021	China	2120	N/A
76	Tsakiridis I [83. ]	2021	Greece	505	N/A
77	Brik M [84. ]	2021	Spain	164	N/A
78	Effati-Daryani F [85. ]	2021	Iran	437	N/A
79	Boekhorst MGBM [86. ]	2021	Netherlands	265	N/A
80	An R [87. ]	2021	China	209	N/A
81	Lubián López DM [88. ]	2021	Spain	514	N/A
82	Maleki A [89. ]	2021	Iran	2336	N/A
83	Khoury JE [90. ]	2021	Canada	304	N/A
84	Suárez-Rico BV [91. ]	2021	Mexico	293	N/A
85	Korukcu O [92. ]	2021	Turkey	497	*p* < 0.0001
86	Obata S [93. ]	2021	Japan	4798	N/A
87	Sakalidis VS [94. ]	2021	Australia and New Zealand	233	N/A
88	Basu A [95. ]	2021	64 countries	6894	N/A
89	Kara P [96. ]	2021	Turkey	445	N/A
90	Fallon V [97. ]	2021	UK	614	p < 0.001
91	Mo PKH [98. ]	2021	China	4087	N/A
92	Wu F [99. ]	2021	Shenzhen	3434	N/A
93	Ding W [100. ]	2021	Wuhan	817	N/A
94	Chrzan-Dętkoś M [101. ]	2021	Poland	78	*p* = 0.025
95	Janevic T [102. ]	2021	New York	228	N/A
96	Thompson KA [103. ]	2021	US	232	N/A
97	Mirzaei N [104. ]	2021	Iran	200	N/A
98	Hiiragi K [105. ]	2021	Japan	279	*p* = 0.17
99	McFarland MJ [106. ]	2021	US	2402	N/A
100	Zhou Y [107. ]	2021	China	1266	N/A
101	Gluska H [108. ]	2021	Israel	421	N/A
102	Liu CH [109. ]	2021	US	628	*p* < 0.01
103	Ramirez Biermann C [110. ]	2021	US	162	N/A
104	Palalioglu RM [111. ]	2021	Turkey	526	N/A
105	Molgora S [112. ]	2020	Italian	389	N/A
106	Patabendige M [113. ]	2020	Sri Lanka	257	N/A
107	Mollard E [114. ]	2021	US	885	N/A
108	Wang J [115. ]	2021	China	2235	N/A
109	Zeng X [116. ]	2020	China	625	N/A
110	Miranda AR MD [117. ]	2021	Argentina	305	N/A
111	Nomura R [118. ]	2021	Brazil	1662	N/A
112	Davis JA [119. ]	2021	US	31	N/A
113	Provenzi L [120. ]	2021	Italy	163	N/A
114	Kotabagi P [121. ]	2020	UK	11	N/A
115	Berthelot N [122. ]	2020	Canada	1258	0.001
116	Corbett GA [123. ]	2020	NA	71	N/A
117	Farrell T [124. ]	2020	Qatar	288	N/A
118	Stepowicz A [125. ]	2020	Poland	210	N/A
119	Mayopoulos GA [126. ]	2021	United States	637	0.008
120	Liu CH [127. ]	2021	United States	1123	N/A
121	Farewell CV [128. ]	2020	United States	27	N/A
122	Haruna M [129. ]	2020	Japan	2872	N/A
123	Bender WR [130. ]	2020	United States	318	N/A
124	Aksoy Derya Y [131. ]	2021	Turkey	48	N/A
125	Nodoushan RJ [132. ]	2020	Iran	560	N/A
126	Mortazavi F [133. ]	2021	Iran	484	N/A
127	Chasson M [134. ]	2021	Israel	233	N/A
128	Taubman-Ben-Ari O [135. ]	2020	Israel	233	N/A
129	Moyer CA [136. ]	2021	Ghana	71	N/A
130	Dib S [137. ]	2020	UK	1329	N/A
131	Qi M [138. ]	2020	China	298	N/A
132	Kassaw C [139. ]	2020	Ethiopia	178	N/A
133	Zheng QX [140. ]	2020	China	331	N/A
134	Machado MMT [141. ]	2021	Brazil	1041	N/A
135	Perzow SED [142. ]	2021	US	135	*p* < 0.001
136	Pope J [143. ]	2021	US,Ireland,UK	573	N/A
137	Kotabagi P [144. ]	2020	UK	14	*p* = 0.9
138	Naurin E [145. ]	2021	Sweden	0	N/A
139	Bo HX [146. ]	2021	China	1309	N/A
140	Barbosa-Leiker C [147. ]	2021	US	162	N/A
141	Stampini V [148. ]	2021	Italy	600	N/A
142	Li C [149. ]	2021	China	2201	N/A
143	Bradfield Z [150. ]	2021	Australia	2840	N/A
144	Kinser PA [151. ]	2021	US	524	N/A
145	Özkan Şat S [152. ]	2021	Turkey	376	N/A
146	Kawamura H [153. ]	2021	Japan	297	N/A
147	Silverio SA [154. ]	2021	UK	710	N/A
148	Ahlers-Schmidt CR [155. ]	2020	US	114	N/A
149	de Arriba-García M [156. ]	2021	Spain	754	N/A
150	Chaves C [157. ]	2021	Spain	724	N/A
151	Wdowiak A [158. ]	2021	Poland	50	N/A
152	Ravaldi C [159. ]	2020	Italy	2448	N/A
153	Wyszynski DF [160. ]	2021	64 countries	7185	N/A
154	Sbrilli MD [161. ]	2021	US	199	N/A
155	Davenport MH [162. ]	2020	Canada	900	*p* < 0.01
156	Di Mascio D [163. ]	2020	China, Saudia Arabia, South Korea, United Arab, Jordan, Canada, USA	19	
157	Juan J [164. ]	2020	USA, Iran, China, Italy, Spain, Peru, Sweden, Turkey, Korea, Australia, Canada and France	24	
158	Amaral WND [165. ]	2020	China, France, US, Iran, Italy, Spain, EUA, Peru, UK, Switzerland, Netherlands, Ireland, Sweden, Canada, Korea	1457	
159	Di Mascio D [166. ]	2020	Argentina, Australia, Belgium, Brazil, Colombia, Czech Republic, Finland, Germany, Greece, Israel, Italy, North Macedonia, Peru, Portugal, Republic of Kosovo, Romania, Russia, Serbia, Slovenia, Spain, Turkey, US	388	
160	Sentilhes L [167. ]	2020	Europe, Sub-Saharan Africa, North Africa	38	
161	Sahin D [168. ]	2021	Turkey	533	
162	Kayem G [169. ]	2020	France	617	
163	Adhikari EH [170. ]	2020	Texas, US	252	
164	Garcia Rodriguez A [171. ]	2020	N/A	1	
165	Islam MM [172. ]	2020	N/A	235	
166	Hansen JN [173. ]	2021	N/A	1	
167	Oltean I [174. ]	2021	N/A	315	
168	Wei SQ [175. ]	2021	N/A	438,548	
169	Singh V [176. ]	2021	India	132	
170	Della Gatta AN [177. ]	2021	China	51	
171	Di Toro F [178. ]	2021	N/A	1104	
172	Bellos I [179. ]	2021	China	158	
173	Abou Ghayda R [180. ]	2020	China,Italy,Iran	104	
174	Remaeus K [181. ]	2020	Sweden	67	
175	Mullins E [182. ]	2020	N/A	1606	
176	Zaigham M [183. ]	2020	China, Sweden, US, Korea, Honduras	108	
177	Yu N [184. ]	2020	China	7	
178	Galang RR [185. ]	2020	N/A	12	
179	Capobianco G [186. ]	2020	N/A	44	
180	Berthelot N [122. ]	2020	Canada	1258	
181	Mappa I [187. ]	2020	Italy	178	
182	Ayaz R [188. ]	2020	N/A	63	
183	Dubey P [189. ]	2020	N/A	790	
184	Pierce-Williams RAM [190. ]	2020	USA	44	
185	Gao YJ [191. ]	2020	N/A	236	
186	Yang R [192. ]	2020	China	65	
187	Yee J [193. ]	2020	N/A	9032	
188	Liu X [194. ]	2020	China	1947	
189	Novoa RH [195. ]	2020	N/A	322	
190	Matar R [196. ]	2020	China, US, Republic of Korea, Honduras	136	
191	Gur RE [197. ]	2020	America	787	
192	Sakowicz A [198. ]	2020	America	1317	
193	Taubman-Ben-Ari O [199. ]	2020	Israel	336	
194	Ng QJ [200. ]	2020	Singapore	324	
195	Hamzehgardeshi Z [201. ]	2020	Iran	318	
196	Ozsurmeli M [202. ]	2020	Turkey	24	
197	Makvandi S [203. ]	2020	N/A	68	
198	Guo Y [204. ]	2020	China	20	
199	Karimi L [205. ]	2020	N/A	571	
200	Waratani M [206. ]	2020	Japan	1	
201	Savasi VM [207. ]	2020	Italy	11	
202	Effati-Daryani F [208. ]	2020	Iran	205	
203	Smith V [209. ]	2020	N/A	92	
204	Chen H [210. ]	2020	China	9	
205	Wang Y [211. ]	2020	China	72	
206	Janevic T [212. ]	2021	USA	3731	
207	Cao D [213. ]	2020	China	10	
208	Lebel C [214. ]	2020	Canada	1764/1757	
209	Marín Gabriel MA [215. ]	2020	Spain	11	
210	Lokken EM [216. ]	2020	America	155	
211	Ashraf MA [217. ]	2020	N/A	90	
212	de Vasconcelos Gaspar A [218. ]	2021	Portugal	7	
213	Huntley BJF [219. ]	2020	N/A	538	
214	Khoury R [220. ]	2020	USA	241	
215	Diriba K [221. ]	2020	N/A	1316	
216	Assiri A [222. ]	2016	N/A	5	
217	Malik A [223. ]	2016	N/A	1	

This table outlines the first author, year of publication, and geographical locations for all studies. Sample size has been recorded and p-value has been included where appropriate

**Table 3:** Studies selected for the meta-analysis

Authors	Country	Sample Size	Publication Year	Symptoms	Measure Name
Lebel C [214. ]	Canada	1757/ 1764	2020	Anxiety,Depression	PROMIS,EPDS
Ayaz R [188. ]	Turkey	63	2020	Anxiety	BAI
Durankuş F [10. ]	Turkey	260	2020	Anxiety,Depression	BAI,EPDS
Liu X [194. ]	China	1947	2020	Anxiety	SAS
Mappa I [187. ]	Italy	178	2020	Anxiety	STAI-T,STAI-S
López-Morales H [66. ]	Argentina	72	2021	Anxiety,Depression	STAI-S,BDI-II
Salehi L [14. ]	Iran	220	2020	Anxiety	CDAS
Gur RE [197. ]	United States	787	2020	Anxiety,Depression	GAD-7,PHQ-2
Ng QJ [200. ]	Singapore	324	2020	Anxiety,Depression,Stress	DASS21-A, DASS21-D, DASS21-S
Effati-Daryani F [208. ]	Iran	205	2020	Anxiety,Depression,Stress	DASS21-A, DASS21-D, DASS21-S
Ravaldi C [17. ]	Italy	200	2021	Anxiety	COVID-ASSESS questionnaire
Zhou Y [18. ]	China	544	2020	Anxiety,Depression,PTSD,Sleep orders	GAD-7,PHQ-9,PCL-5,ISI
Kahyaoglu Sut H [19. ]	Turkey	403	2021	Anxiety,Depression	HADS-A, HADS-D
Sinaci S [22. ]	Turkey	200	2020	Anxiety	STAI-T,STAI-S
Dong H [23. ]	China	156	2021	Anxiety,Depression	SAS,SDS
Hocaoglu M [24. ]	Turkey	283	2020	Anxiety,PTSD	STAI-T,STAI-S/IES-R
Yue C [27. ]	China	308	2021	Anxiety	SAS
Taubman-Ben-Ari O [199. ]	Israel	336	2020	Anxiety	self-designed questionnaire
Maharlouei N [28. ]	Iran	540	2020	Anxiety	self-designed questionnaire
Milne SJ [30. ]	Ireland	70	2020	Anxiety	N/A
Ceulemans M [32. ]	Ireland, Norway, Switzerland, the Netherlands, and the UK	3545	2021	Anxiety,Depression,Stress	GAD-7,EDS, PSS-10
Yassa M [35. ]	Turkey	203	2020	Anxiety	STAI-S,STAI-T
Jiang H [39. ]	China	1873	2021	Anxiety,Depression,Stress	SAS,EDS, CPSS-14
Mayeur A [41. ]	France	88	2020	Anxiety	self-designed questionnaire
Lin W [42. ]	China	751	2021	Anxiety,Depression	SAS,PHQ-9
Yang X [44. ]	Chinese	19,515	2021	Anxiety,Depression	GAD-7,PHQ-9
Akgor U [48. ]	Turkey	297	2021	Anxiety,Depression	HADS-A, HADS-D
Preis H [50. ]	US	788/4451	2020	Anxiety,Stress	GAD-7,PREPS
Dagklis T [51. ]	Greece	269/215	2020	Anxiety,Depression	STAI-S,STAI-T/EPDS
Esteban-Gonzalo S [53. ]	Spain	353	2021	Anxiety	STAI-S
Koyucu RG [54. ]	Turkey	729	2021	Anxiety,Depression,Stress	DASS21-A, DASS21-D, DASS21-S
Liu J [58. ]	US	715	2021	Anxiety,Depression	GAD-7,EPDS
Cao Y [60. ]	China	298	2021	Anxiety,Depression	N/A
Mappa I [61. ]	Italy	161	2021	Anxiety	STAI-T,STAI-S
Mehdizadehkashi A [62. ]	Iran	300	2021	Anxiety	self-designed questionnaire
Yirmiya K [63. ]	Israel	1114	2021	Anxiety,Depression,Stress	GAD-7,PHQ-2,PREPS
Xie M [64. ]	China	689	2021	Anxiety,Depression,Sleep disorders	SCL90-R,PSQI
Ge Y [65. ]	China	446	2021	Anxiety	SAS
López-Morales H [66. ]	Argentina	102	2021	Anxiety,Depression	STAI-S,BDI-II
Puertas-Gonzalez JA [67. ]	Spain	100	2021	Anxiety,Depression,Stress	SCL-90-R,PSS-14
Çolak S [68. ]	Turkey	149	2021	Anxiety,Depression,Stress	BAI,BDI,PSQI
Xu K [69. ]	China	274	2021	Anxiety,Depression,Stress,Sleep disorders	SAS,EPDS,CPSS,PSQI
Zilver SJM [70. ]	Netherlands	1102	2021	Anxiety,Depression,Stress	HADS-A,HADS-D,PSS-10
Maharlouei N [71. ]	Iran	540	2021	Anxiety,Depression,Stress	DASS21-A, DASS21-D, DASS21-S
Harrison V [72. ]	UK	205	2021	Anxiety,Depression	PASS,EPDS
Saadati N [73. ]	Iran	300	2021	Anxiety	HAQ
Wang Q [74. ]	China	15,428	2021	Anxiety,Depression	GAD-7,PHQ-9
Behmard V [75. ]	Iran	801	2021	Anxiety	CDAS
Hamzehgardeshi Z [201. ]	Iran	318	2021	Anxiety,Depression	PRAQ,EPDS
Jelly P [78. ]	India	333	2021	Anxiety	GAD-7
Wang Q [74. ]	China	19,515	2021	Anxiety,Depression	GAD-7,PHQ-9
Zhang Y [80. ]	China	1794/560	2021	Anxiety,Stress	SAS,IES
Masjoudi M [81. ]	Iran	215	2021	Anxiety,Stress	CDAS,PSS-14
Shangguan F [82. ]	China	2120	2021	Anxiety,Stress	GAD-7,PSS
Tsakiridis I [83. ]	Greece	505	2021	Anxiety,Depression	STAI-S,STAI-T/EPDS
Brik M [84. ]	Spain	109/164	2021	Anxiety	STAI-S,STAI-T/EPDS
Effati-Daryani F [85. ]	Iran	437	2021	Anxiety,Depression,Stress	DASS21-A, DASS21-D, DASS21-S
Lubián López DM [88. ]	Spain	514	2021	Anxiety	STAI-S,STAI-T/EPDS
Maleki A [89. ]	Iran	2336	2021	Anxiety	GAD-7
Khoury JE [90. ]	Canada	304	2021	Anxiety,Depression,Stress,Sleep disorders	GAD-7,CES-D,PSS-10,ISI
Suárez-Rico BV [91. ]	Mexico	293	2021	Anxiety	STAI-T
Obata S [93. ]	Japan	4798	2021	Anxiety,Depression	K6,EPDS
Mo PKH [98. ]	China	4087	2021	Anxiety,Depression	GAD-7,PHQ-9
Wu F [99. ]	Shenzhen	3434	2021	Anxiety,Depression	GAD-7,PHQ-9
Ding W [100. ]	Wuhan	817	2021	Anxiety	SAS
Mirzaei N [104. ]	Iran	200	2021	Anxiety,Depression	HADS-A, HADS-D
Ramirez Biermann C [110. ]	US	162	2021	Anxiety,Depression	self-designed questionnaire
Palalioglu RM [111. ]	Turkey	526	2021	Anxiety	self-designed questionnaire
Molgora S [112. ]	Italian	389	2020	Anxiety,Depression	STAI-S,STAI-T/EPDS
Patabendige M [113. ]	Sri Lanka	257	2020	Anxiety,Depression	HADS-A, HADS-D
Zeng X [116. ]	China	516	2020	Anxiety,Depression,Sleep disorders	GAD-7,EPDS, DSM-IV
Nurrizka RH [77. ]	Indonesia	36	2021	Anxiety	DASS-21-A
Wu Y [9. ]	China	1285	2020	Depression	EPDS
Wang Y [211. ]	China	72	2020	Depression,PTSD	EPDS,PCL-C
Medina-Jimenez V [29. ]	Mexico	503	2020	Depression,Stress	EPDS,PSS
Matsushima M [31. ]	Japan	1777	2020	Depression	EPDS
Gildner TE [33. ]	US	1856	2020	Depression	EPDS
Shayganfard M [34. ]	Iran	66	2020	Depression,Stress	EPDS,PSS-14
Silverman ME [36. ]	US	516	2020	Depression	EPDS
Muhaidat N [37. ]	Jordan	944	2020	Depression	self-designed questionnaire
Thayer ZM [38. ]	US	2099	2021	Depression	EPDS
Zhang CJP [43. ]	China	1901	2020	Depression,PTSD	EPDS,PCL-S
Khamees RE [45. ]	Egypt	120	2021	Depression	EPDS
Silverman ME [47. ]	US	485	2020	Depression	EPDS
Shahid A [49. ]	Pakistan	552	2020	Depression,Sleep disorders	EPDS,self-designed questionnaire
Ionio C [52. ]	Italy	75	2021	Depression	EPDS
Overbeck G [55. ]	Denmark	330	2021	Depression	MDI
Kachi Y [56. ]	Japan	270	2021	Depression	EPDS
Smith CL [59. ]	USA	83	2021	Depression,Stress	EPDS,PSS-10
King LS [76. ]	US	725	2021	Depression	EPDS
Korukcu O [92. ]	Turkey	497	2021	Depression	EDS
Zhou Y [107. ]	China	1266	2021	Depression	PHQ-9
Chaves C [157. ]	Spain	450	2021	Depression	EPDS
Davis JA [119. ]	US	31	2021	Stress	PSS-10
Ionio C [52. ]	Italy	75	2021	PTSD	IES-R
Basu A [95. ]	64 countries	5712	2021	PTSD	IES-6
Kara P [96. ]	Turkey	445	2021	PTSD	PCL-5
Wang J [115. ]	China	2235	2021	Sleep disorders	ISI

This table outlines first author, year of publication, and geographical location for the studies. Sample size, mental health symptoms and names of measures were included

#### Study design, source of data, data collection method and sample size

All 217 studies used different study designs; 107 cross-sectional, 7 cohort and 7 case controlled. A total of 23 qualitative studies used self-reported methods of data collection. Real-world data from hospital admissions were used in 5 studies whilst 2 extracted data from patient medical records. The 217 study-pool comprised of a sample of 638,889 pregnant women, including 6898 women who were within 90 days of delivery. The sample sizes used within the studies varied considerably; 129 studies comprised of approximately 500 women, 40 studies consisted of500–999 ladies, 18 studies had 1000–1999 women and 24 studies had ≥2000 women.

#### Stages of pregnancy assessed

A total of 99 studies reported pregnant women during their first, second and third trimester.

#### Site of data collection

Many studies reported that data collection took place during routine antenatal or postnatal visits in outpatient departments, tertiary/provincial hospitals, secondary level or district hospitals, and primary healthcare facility level.

#### Mental health outcomes assessed

Sixty-four reported data on depressive symptoms, 82 on symptoms of anxiety, 20 on symptoms of stress, 7 on PTSD symptoms, and 8 on symptoms of sleep disorders. Detailed characteristics of the systematically included studies and those meta-analysed are listed in Tables 1 and 2 (see page 33 for Table 1 and page 35 for Table 2).

### Meta-analysis

#### Depression

**Fig. 2:**
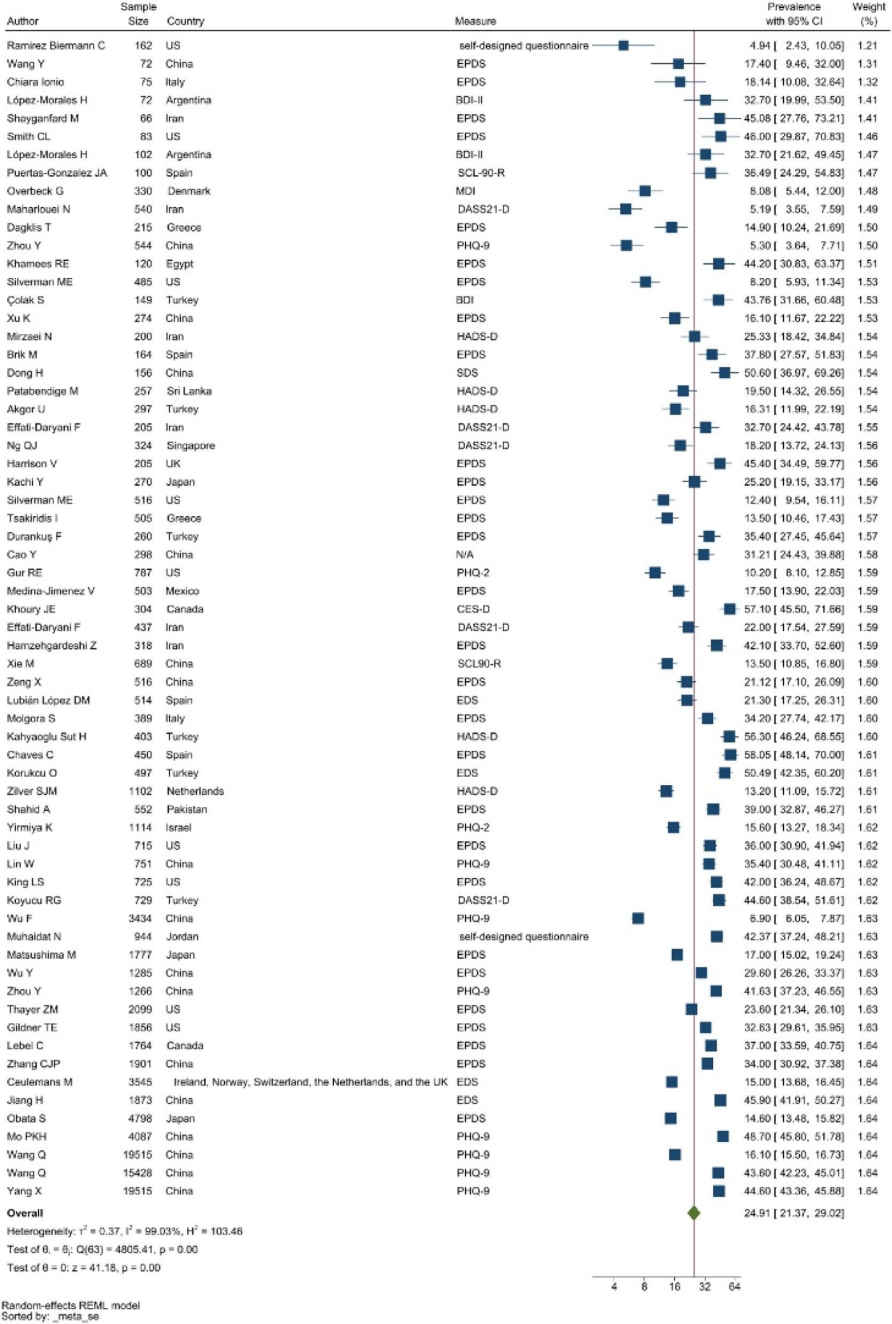
Forest plot showing prevalence of depressive symptoms. Legend. The forest plot shows the first author, sample size and geographic location for the included studies. Measure of depressive symptoms, prevalence rate with confidence internal and weighting of results have also been displayed

Edinburgh Postnatal Depression Scale (EPDS), the Patient Health Questionnaire 9-item (PHQ-9), and the depression subscale of the Hospital Anxiety and Depression Scale (HADS-D) were the commonly used data collection tools to assess symptoms of depression. The pooled prevalence of depression was 24.91% with a 95% CI of 21.37–29.02% (see Fig. 2).

#### Anxiety

**Fig. 3:**
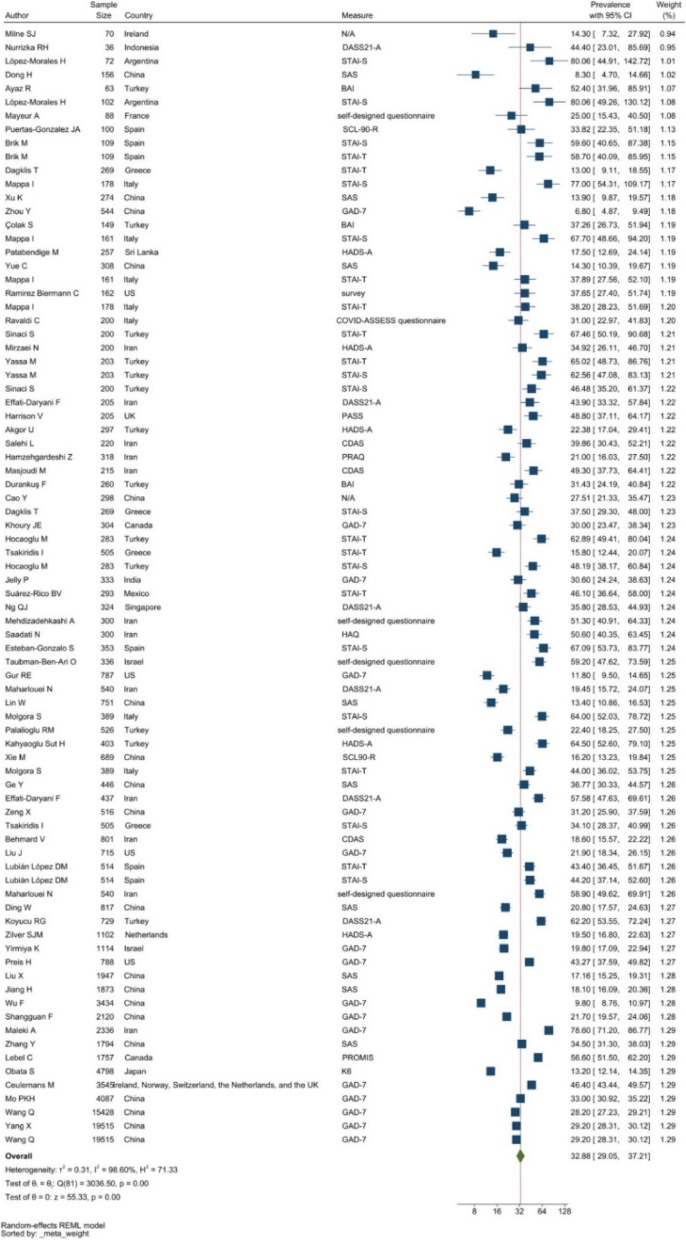
Forest plot showing prevalence of anxiety symptoms. Legend. The above forest plot shows the first author, sample size and geographic location for the included studies. Measure of anxiety symptoms, prevalence rate with confidence internal and weighting of results have also been displayed

Anxiety symptoms were commonly measured by the State-Trait Anxiety Inventory (STAI, with two subscales STAI-T and STAI-S), the General Anxiety Disorder 7-item (GAD-7), and Self-rating Anxiety Scale (SAS). Anxiety prevalence was 32.88% with a 95% CI of 29.05 to 37.21% (see Fig. 3).

#### Str ess

**Figure 4:**
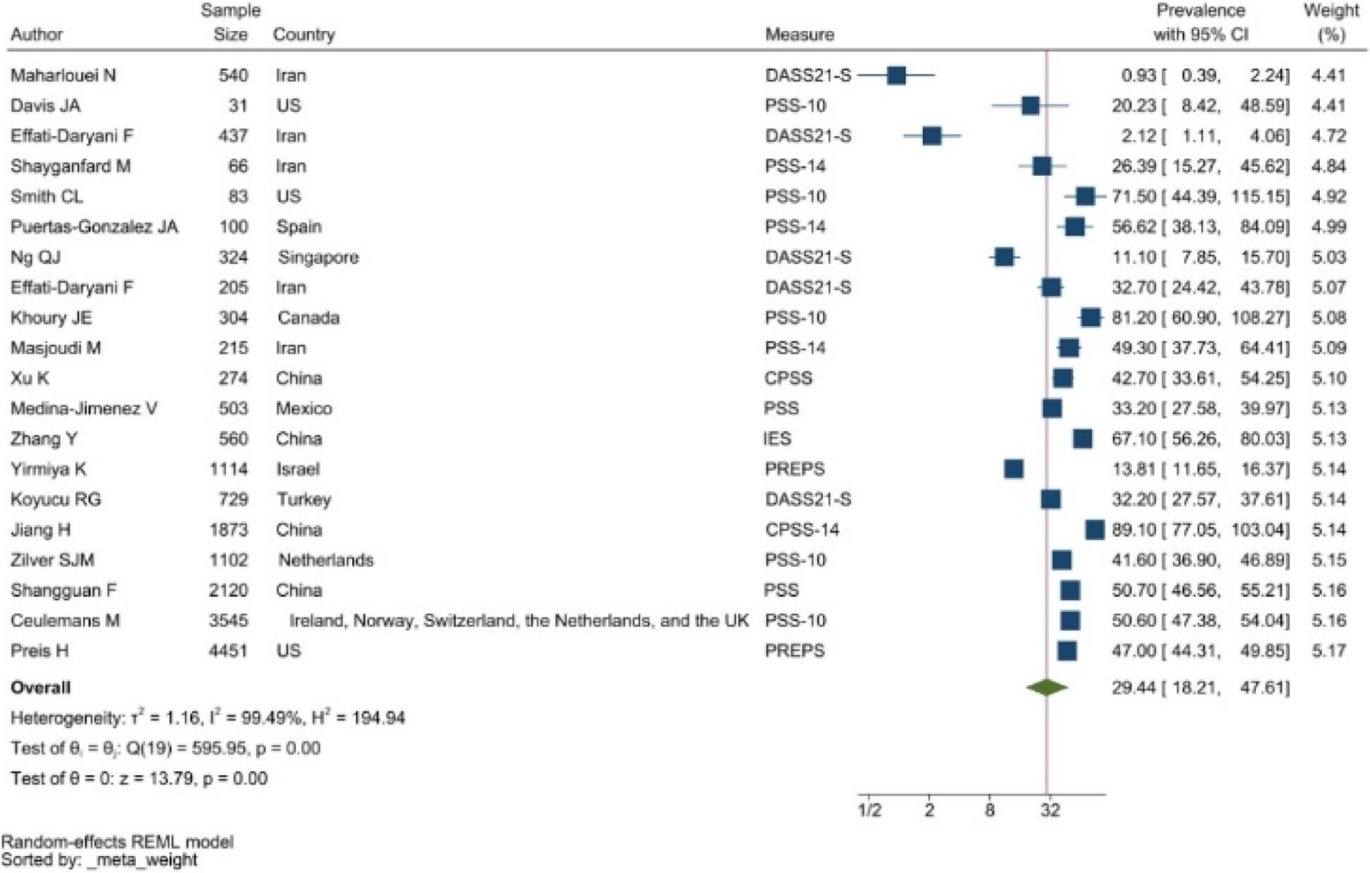
Forest plot showing prevalence of stress symptoms. Legend. The above forest plot shows the first author, sample size and geographic location for the included studies. Measure of stress symptoms, prevalence rate with confidence internal and weighting of results have also been displayed

Tools like the Perceived Stress Scale (PSS, with 10-item and 14-item versions), and the stress subscale of the 21-item Depression Anxiety and Stress Scale (DASS21-S) were frequently used to evaluate stress symptoms. The pooled prevalence of stress among perinatal women was 29.44% (95% CI: 18.21–47.61%) (see Fig. 4).

#### Post-traumatic stress disorder

**Fig. 5:**
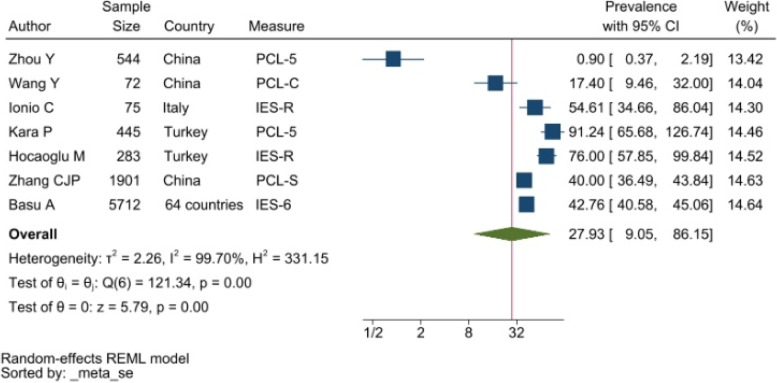
Forest plot showing symptoms of PTSD. Legend. The above forest plot shows the first author, sample size and geographic location for the included studies. Measure of PTSD symptoms, prevalence rate with confidence internal and weighting of results have also been displayed

PTSD symptoms were typically measured by the DSM-V Post-Traumatic Stress Disorder Checklist (PCL-5) and the Impact of Events Scale (IES). The studies reporting PTSD symptoms were heterogeneous resulting in a pooled prevalence of 27.93% with a 95%CI of 9.05–86.15% (see Fig. 5).

#### Insomnia

**Fig. 6:**
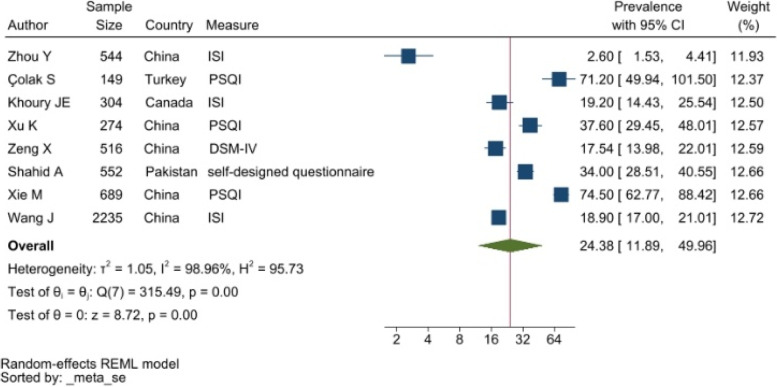
Forest plot showing symptoms of sleep disorders. Legend. The above forest plot shows the first author, sample size and geographic location for the included studies. Measure of sleep disorder symptoms, prevalence rate with confidence internal and weighting of results have also been displayed

The Insomnia Severity Index (ISI) and the Pittsburgh Sleep Quality Index (PSQI) were to assess and report symptoms associated with sleep disorders. The pooled prevalence was 24.38% with a 95% CI of 11.89–49.96% (see Fig. 6).

### Subgroup analysis

The I^2^ evaluated for symptoms of depression, anxiety, PTSD, stress, and sleep disorders were over 98%, which demonstrates a high heterogeneity among the studies. Therefore, a subgroup analysis was conducted to further evaluate the heterogeneity. To determine the symptom prevalence, women were assessed at different stages of their pregnancy and the dataset was categorised based on the trimesters:1st trimester (< 12 weeks), 2nd trimester (13–27 weeks), 3rd trimester (28–41 weeks)] and the immediate post-partum period (immediately after childbirth and up to 6 weeks) for studies that reported follow-up details.

**Fig. 7:**
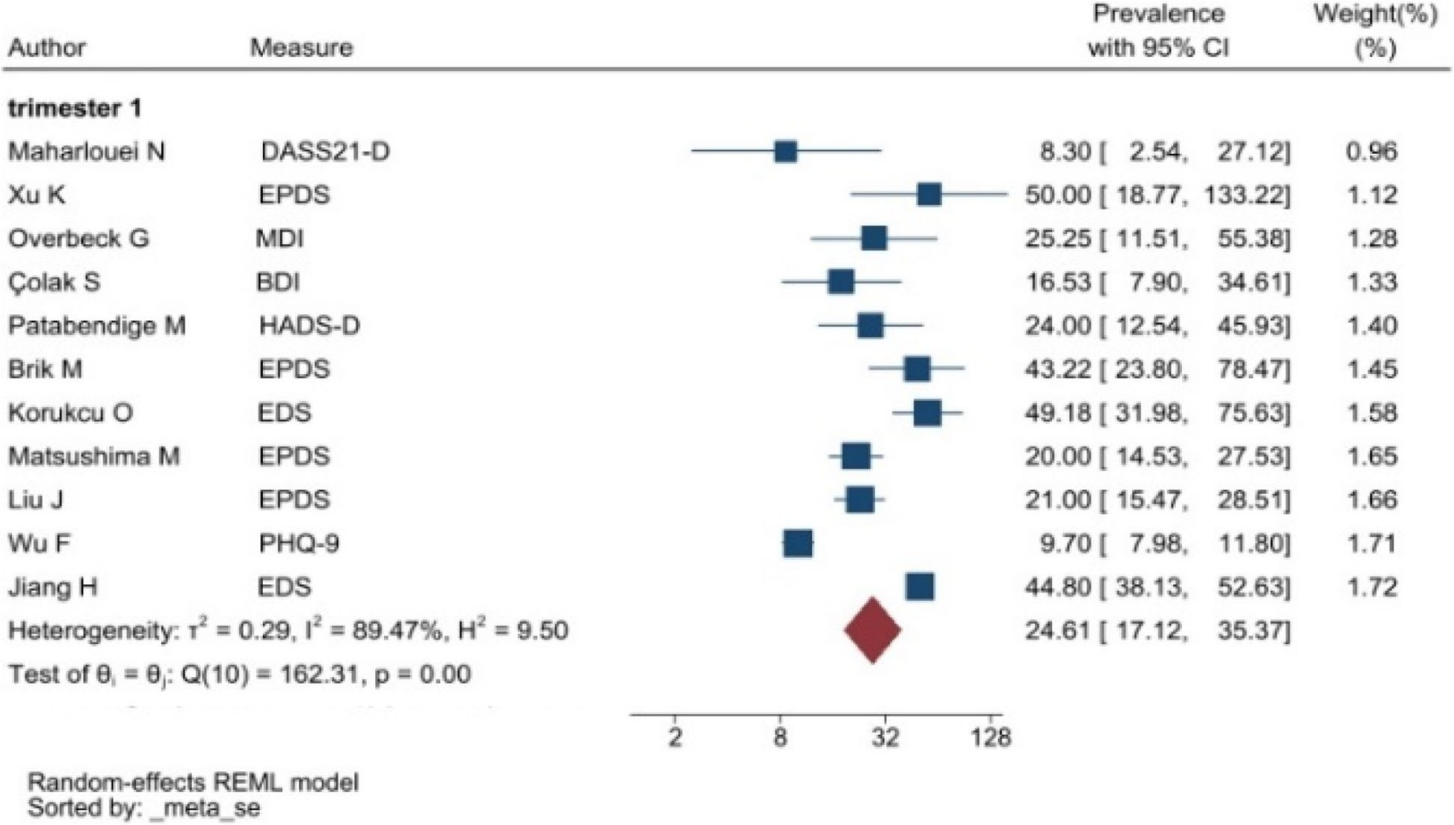
A forest plot showing the subgroup analysis for depressive symptoms in the 1st trimester. Legend. First author and outcomes measures have been included. Prevalence rate with confidence internal and weighting of results have been shown

**Fig. 8:**
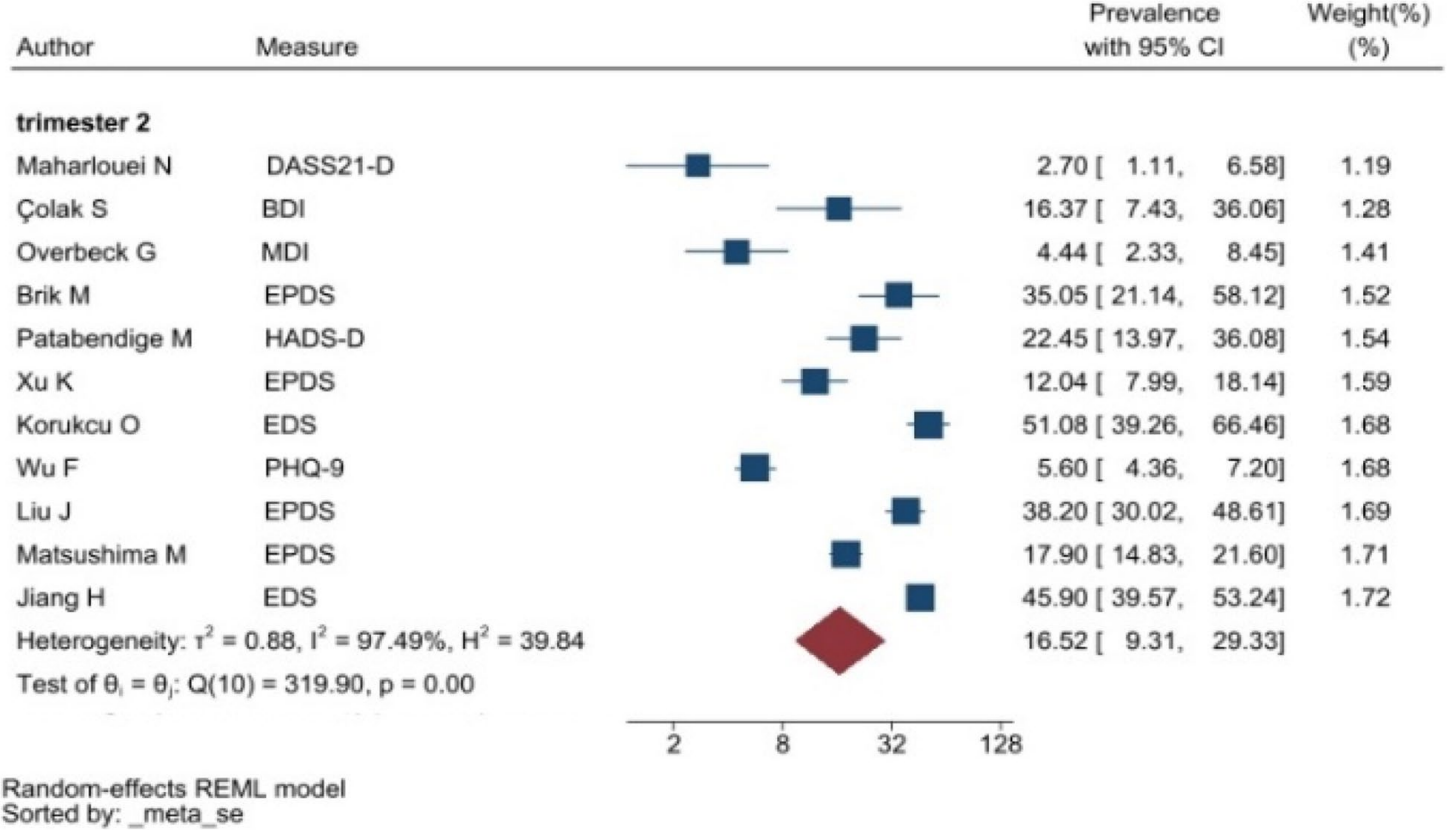
A forest plot showing the subgroup analysis for depressive symptoms in the 2nd trimester. Legend. First author and outcomes measures have been included. Prevalence rate with confidence internal and weighting of results have been shown

**Fig. 9:**
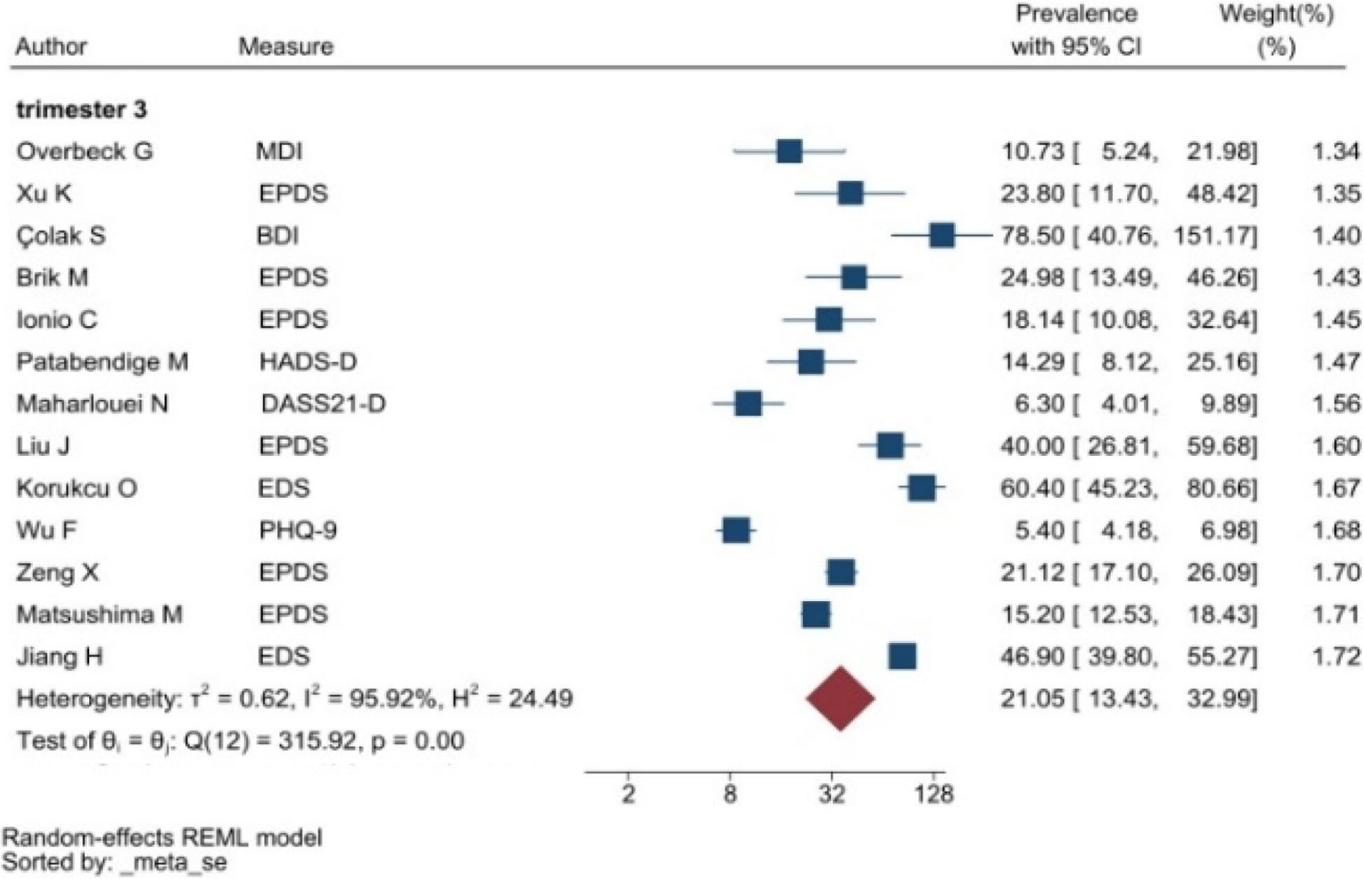
A forest plot showing the subgroup analysis for depressive symptoms in the 3rd trimester. Legend. First author and outcomes measures have been included. Prevalence rate with confidence internal and weighting of results have been shown

**Fig. 10:**
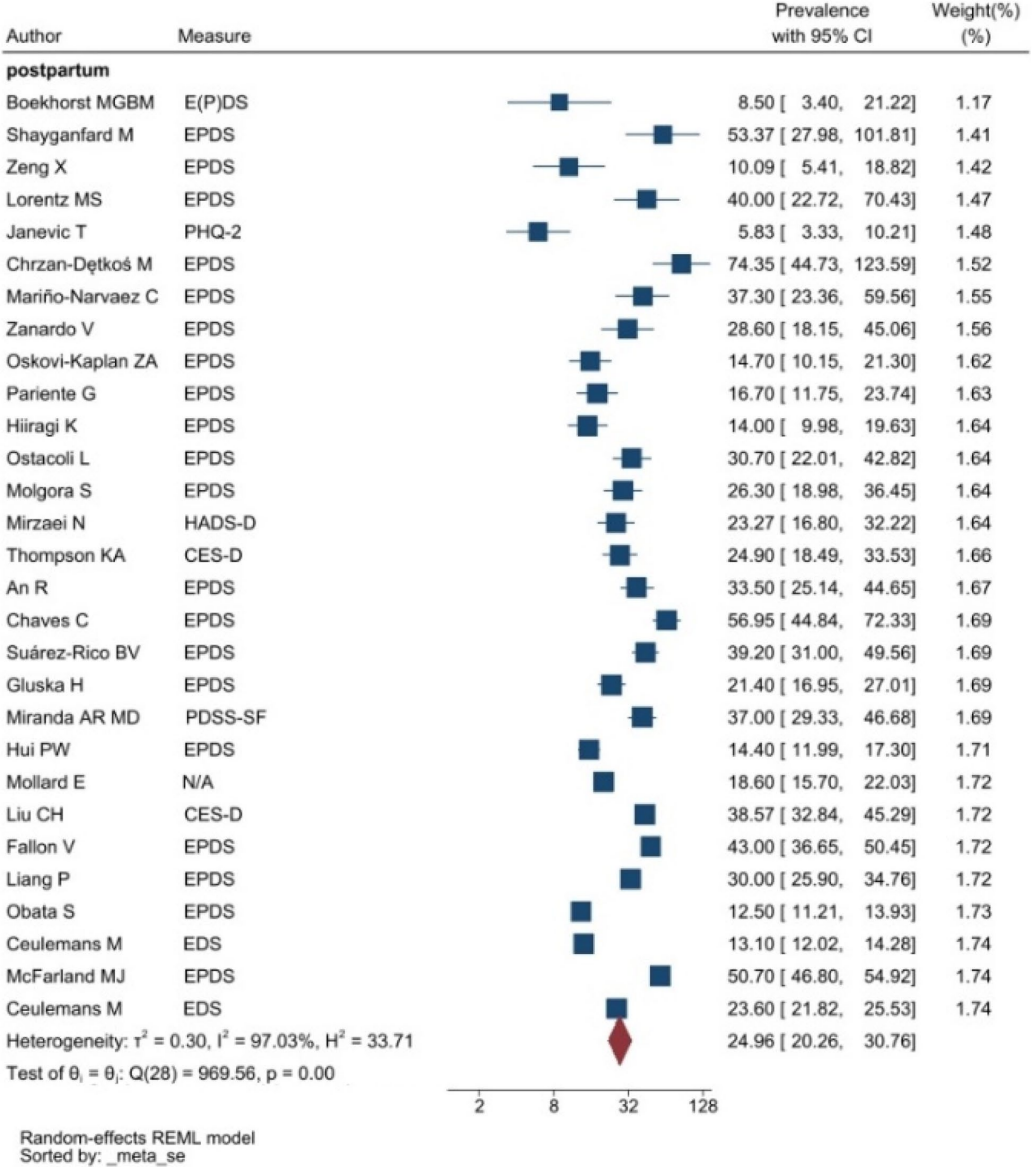
A forest plot showing the subgroup analysis for depressive symptoms postpartum. Legend. First author and outcomes measures have been included. Prevalence rate with confidence internal and weighting of results have been shown

**Fig. 11:**
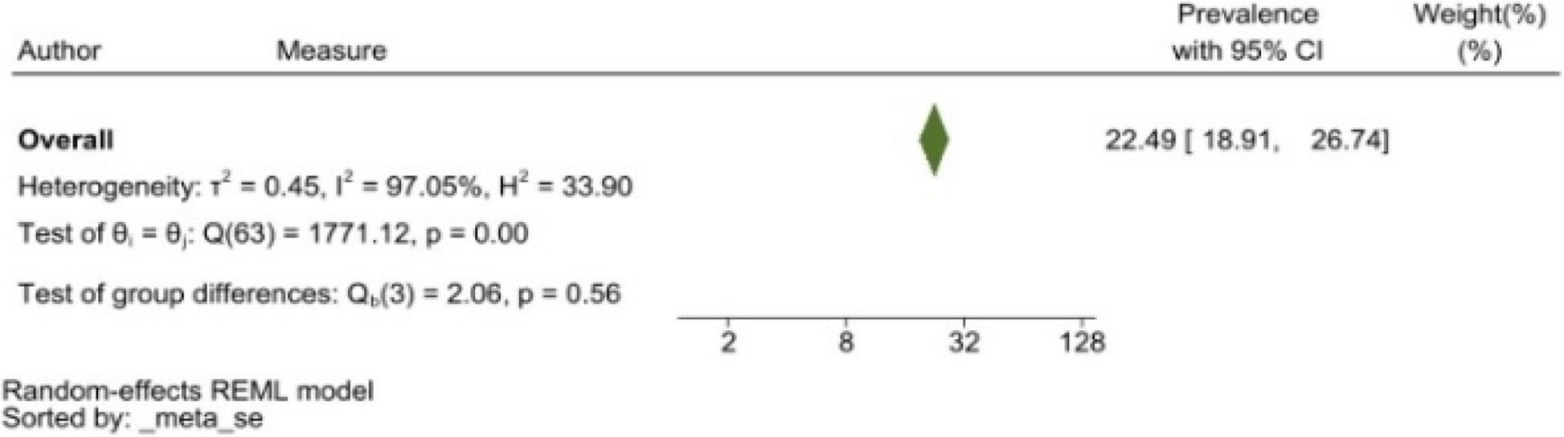
A forest plot showing the overall subgroup analysis for depressive symptoms. Legend. First author and outcomes measures have been included. Prevalence rate with confidence internal and weighting of results have been shown

**Fig. 12:**
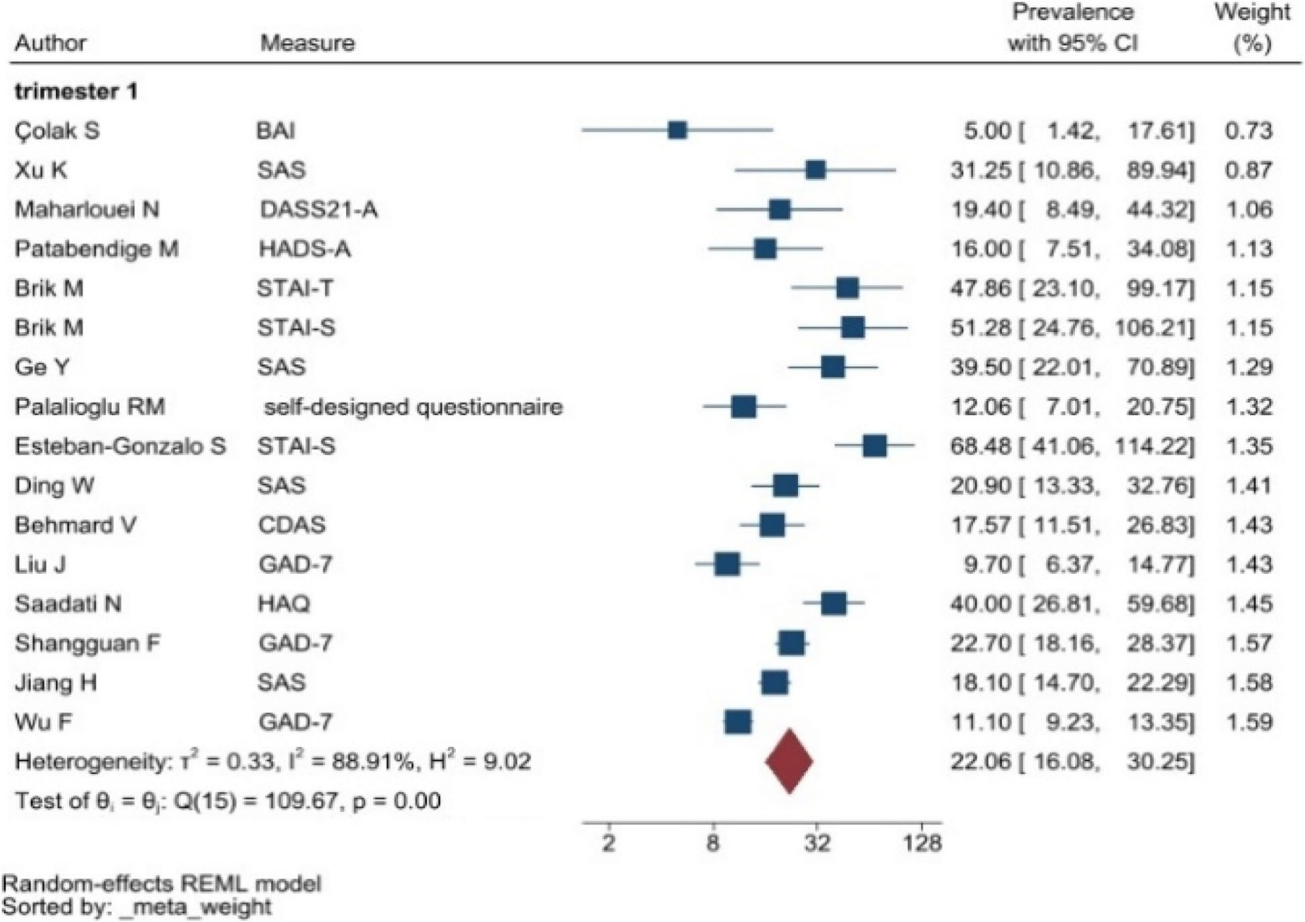
A forest plot showing the subgroup analysis for anxiety symptoms in the 1st trimester. Legend First author and outcomes measures have been included. Prevalence rate with confidence internal and weighting of results have been shown

**Fig. 13:**
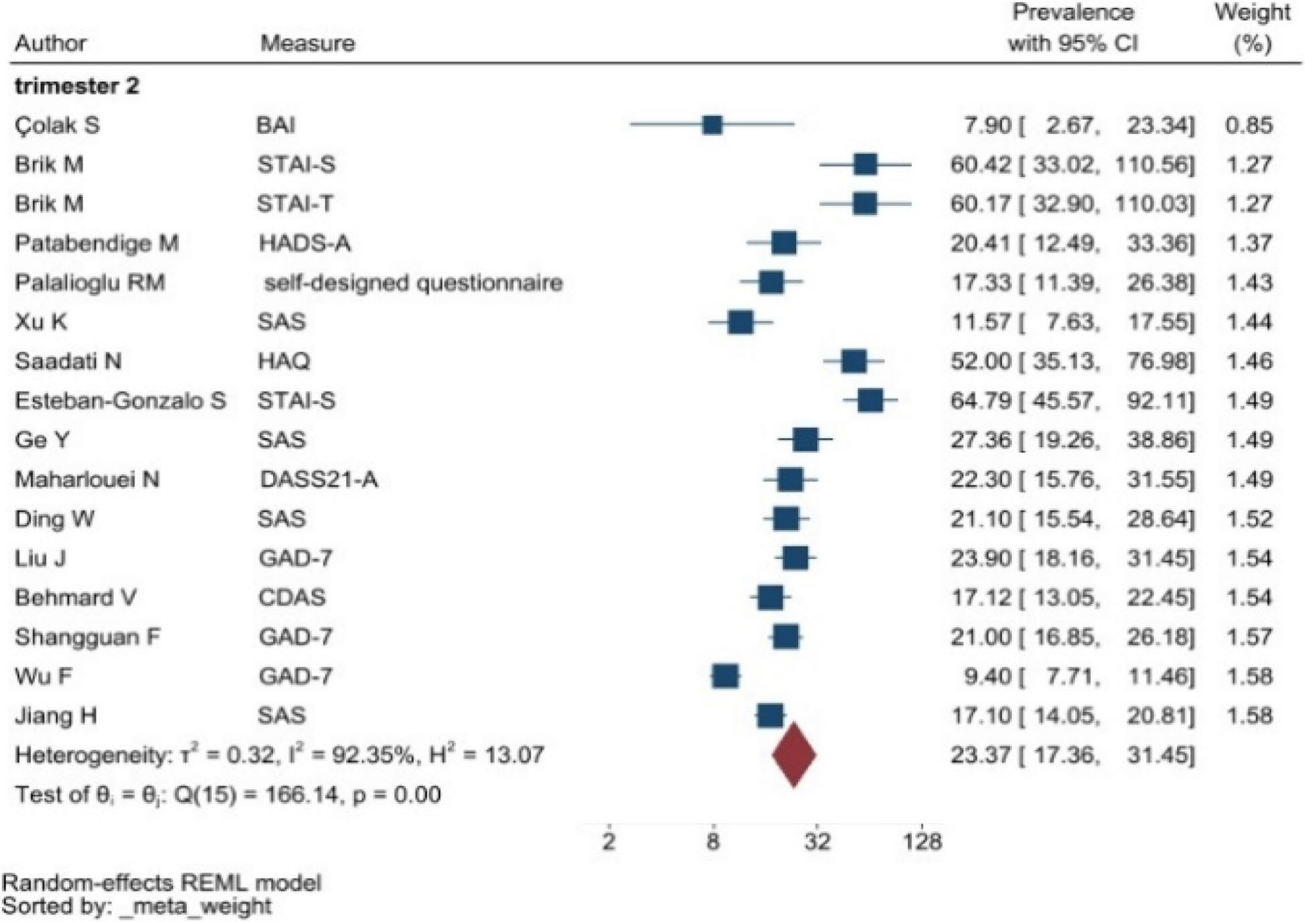
A forest plot showing the subgroup analysis for anxiety symptoms in the 2nd trimester. Legend. First author and outcomes measures have been included. Prevalence rate with confidence internal and weighting of results have been shown

**Fig. 14:**
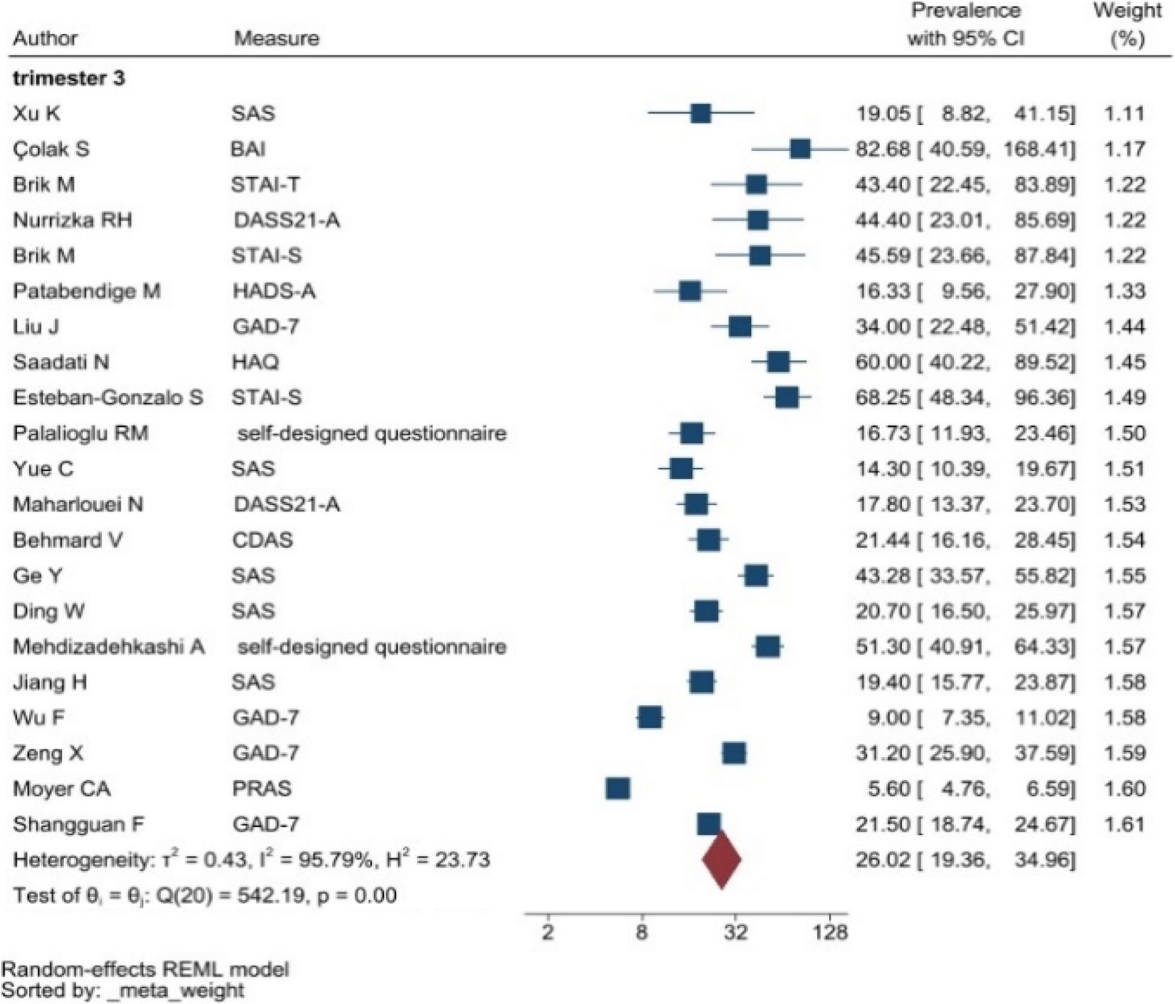
A forest plot showing the subgroup analysis for anxiety symptoms in the 3rd trimester. Legend. First author and outcomes measures have been included. Prevalence rate with confidence internal and weighting of results have been shown

**Fig. 15:**
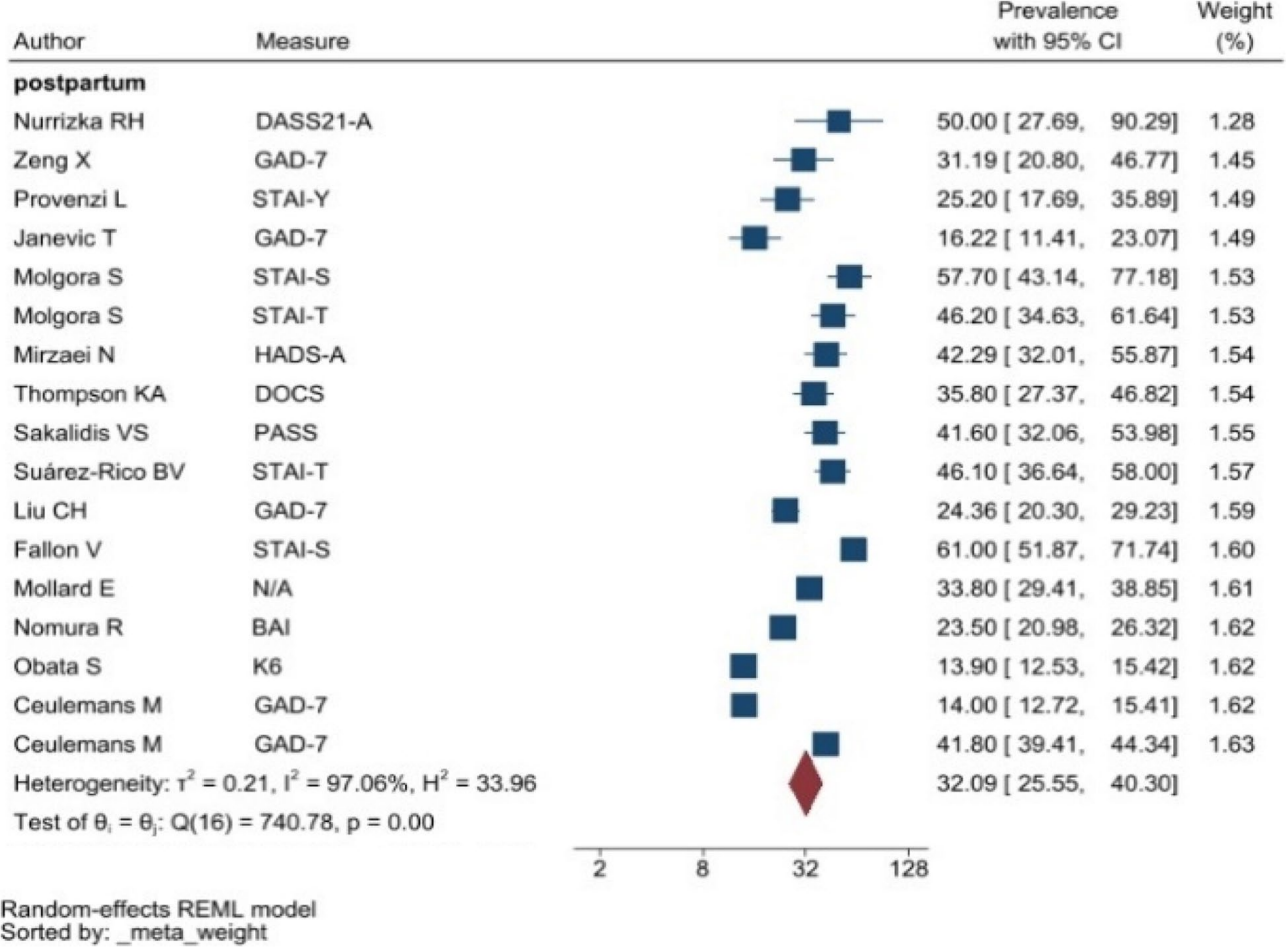
A forest plot showing the subgroup analysis for anxiety symptoms postpartum. Legend. First author and outcomes measures have been included. Prevalence rate with confidence internal and weighting of results have been shown

**Fig. 16:**
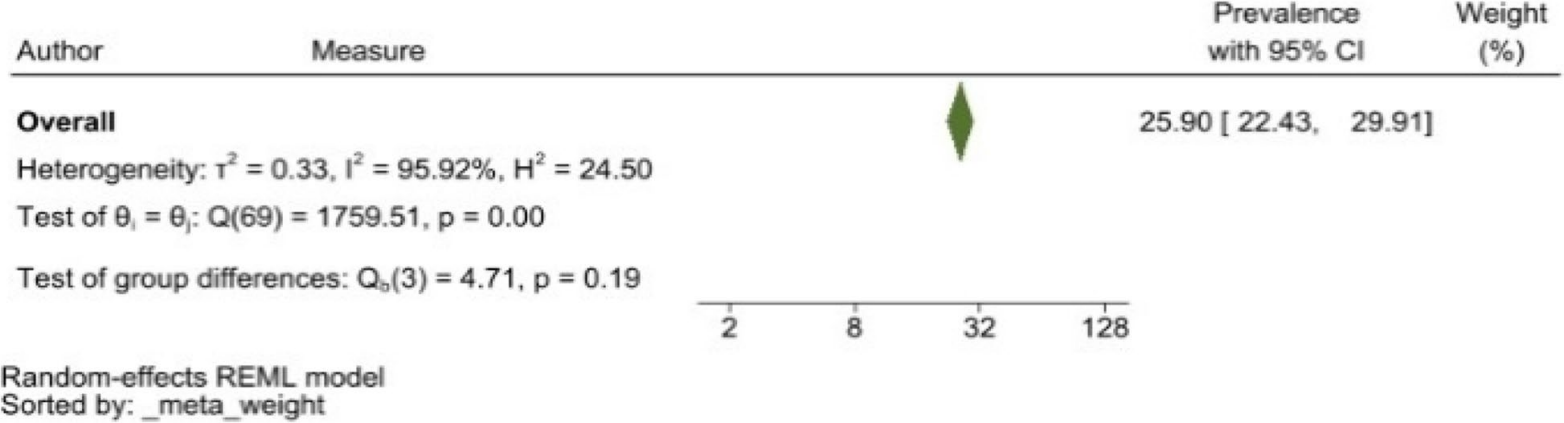
A forest plot showing the overall subgroup analysis for anxiety symptoms. Legend First author and outcomes measures have been included. Prevalence rate with confidence internal and weighting of results have been shown

**Fig. 17:**
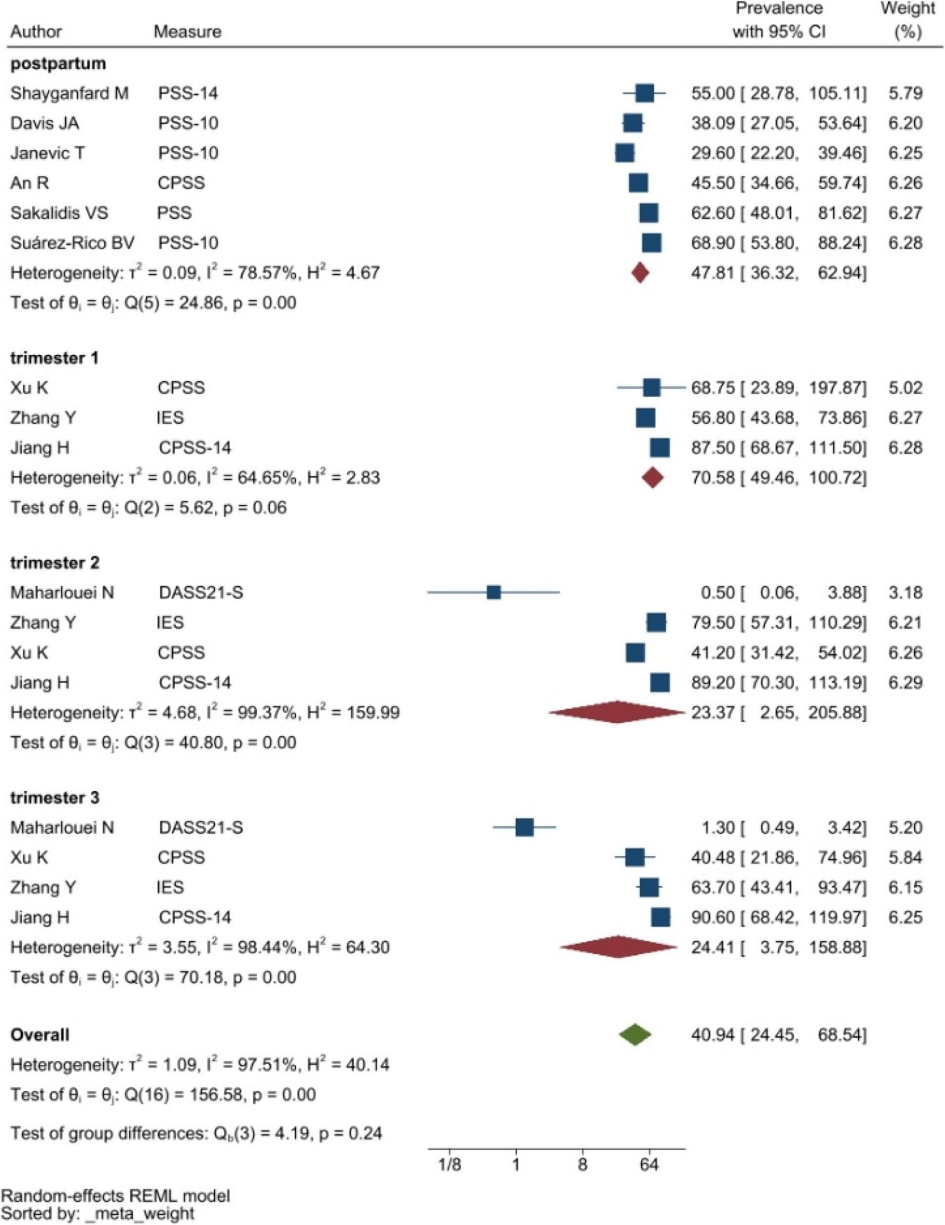
A forest plot showing the subgroup analyses for stress symptoms. Legend. The forest plot shows the subgroup analyses results during the 1st, 2nd, and 3rd trimester as well as postpartum and overall. First author and outcomes measures have been included. Prevalence rate with confidence internal and weighting of results have been shown

**Fig. 18:**
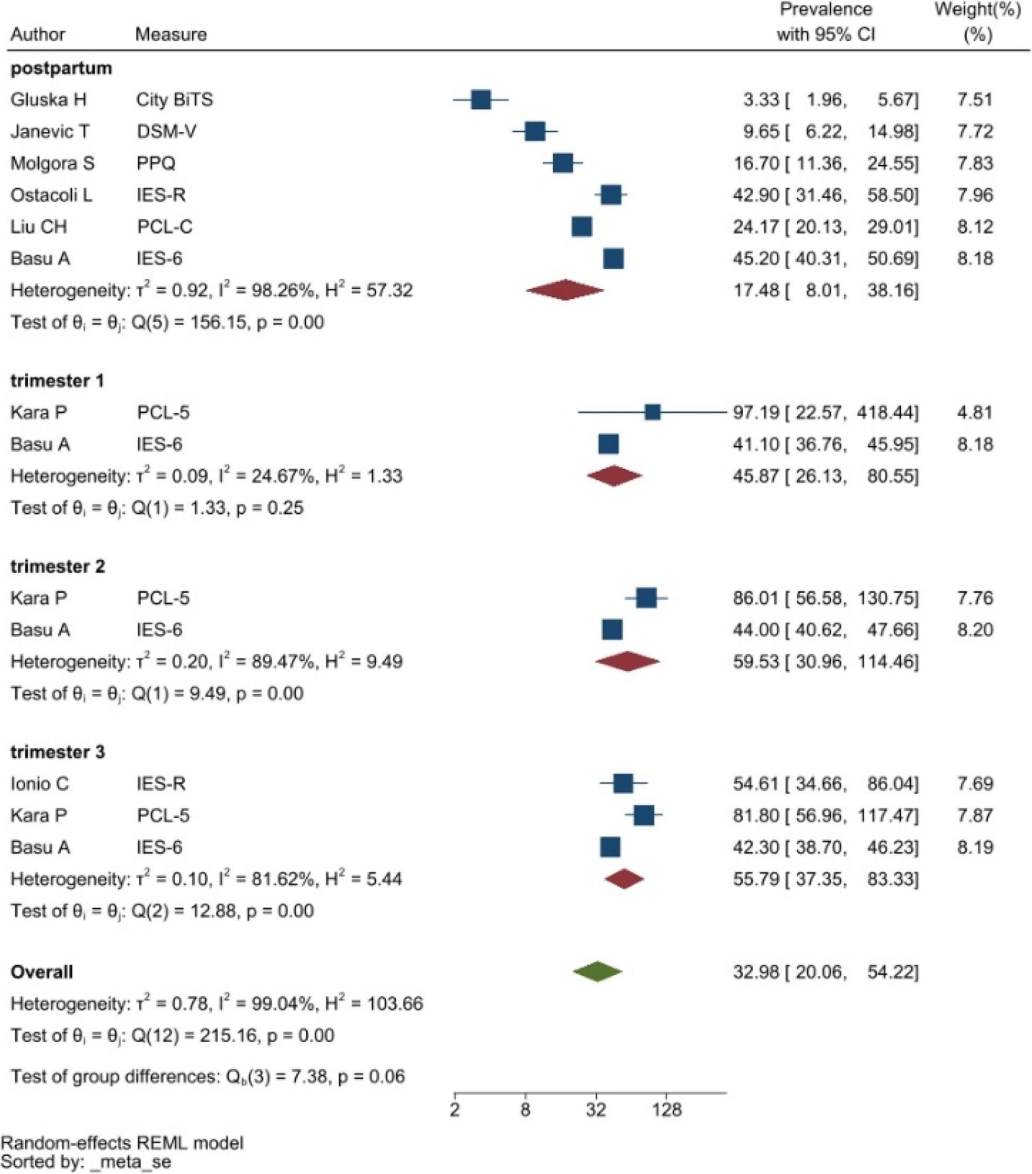
A forest plot showing the subgroup analyses for PTSD symptoms. Legend. The forest plot shows the subgroup analyses results during the 1st, 2nd, and 3rd trimester as well as postpartum and overall. First author and outcomes measures have been included. Prevalence rate with confidence internal and weighting of results have been shown

**Fig. 19:**
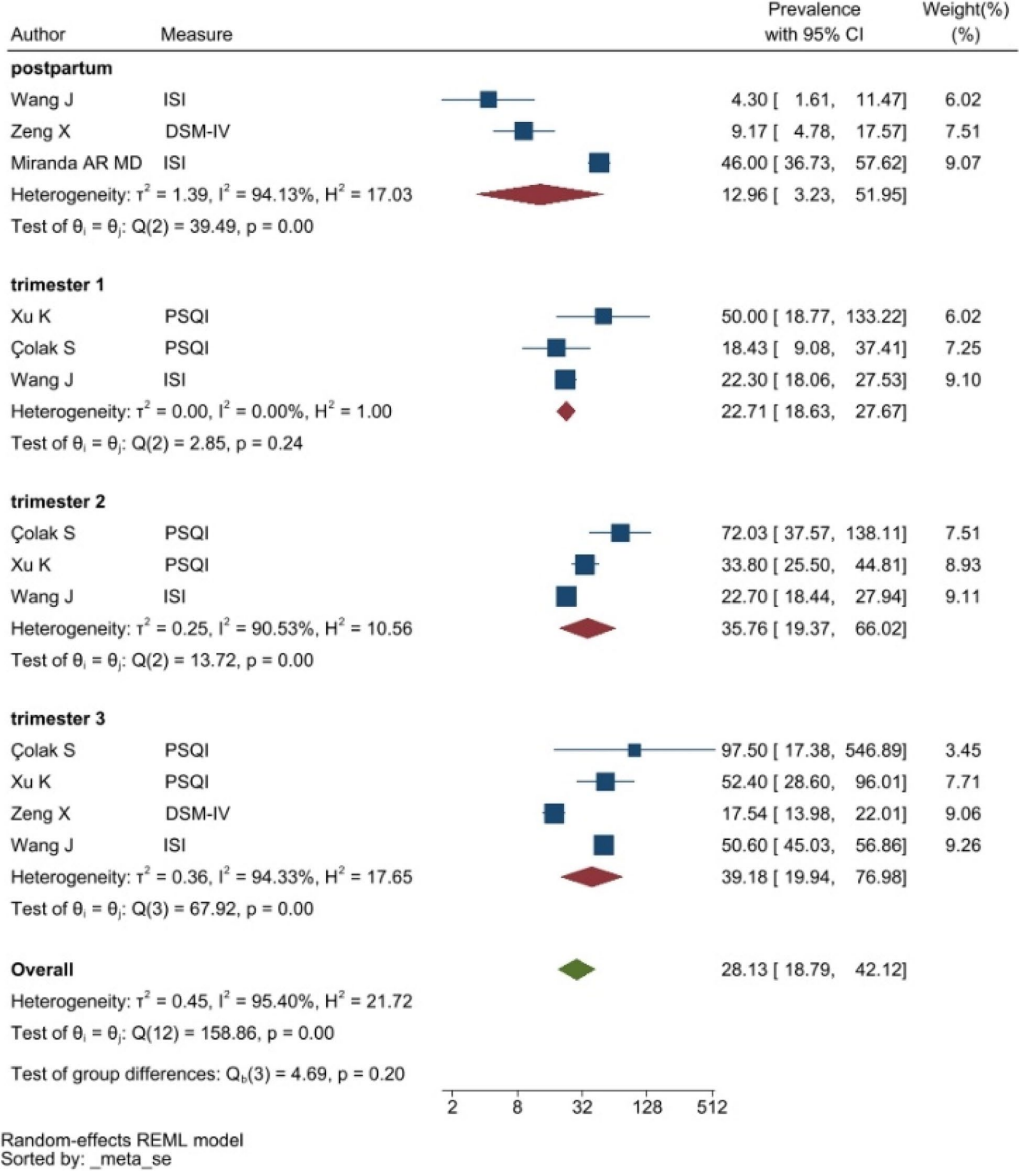
A forest plot showing the subgroup analyses for sleep disorder symptoms. Legend. The forest plot shows the subgroup analyses results for sleep disorder symptoms during the 1st, 2nd, and 3rd trimester as well as postpartum and overall. First author and outcomes measures have been included. Prevalence rate with confidence internal and weighting of results have been shown

**Fig. 20:**
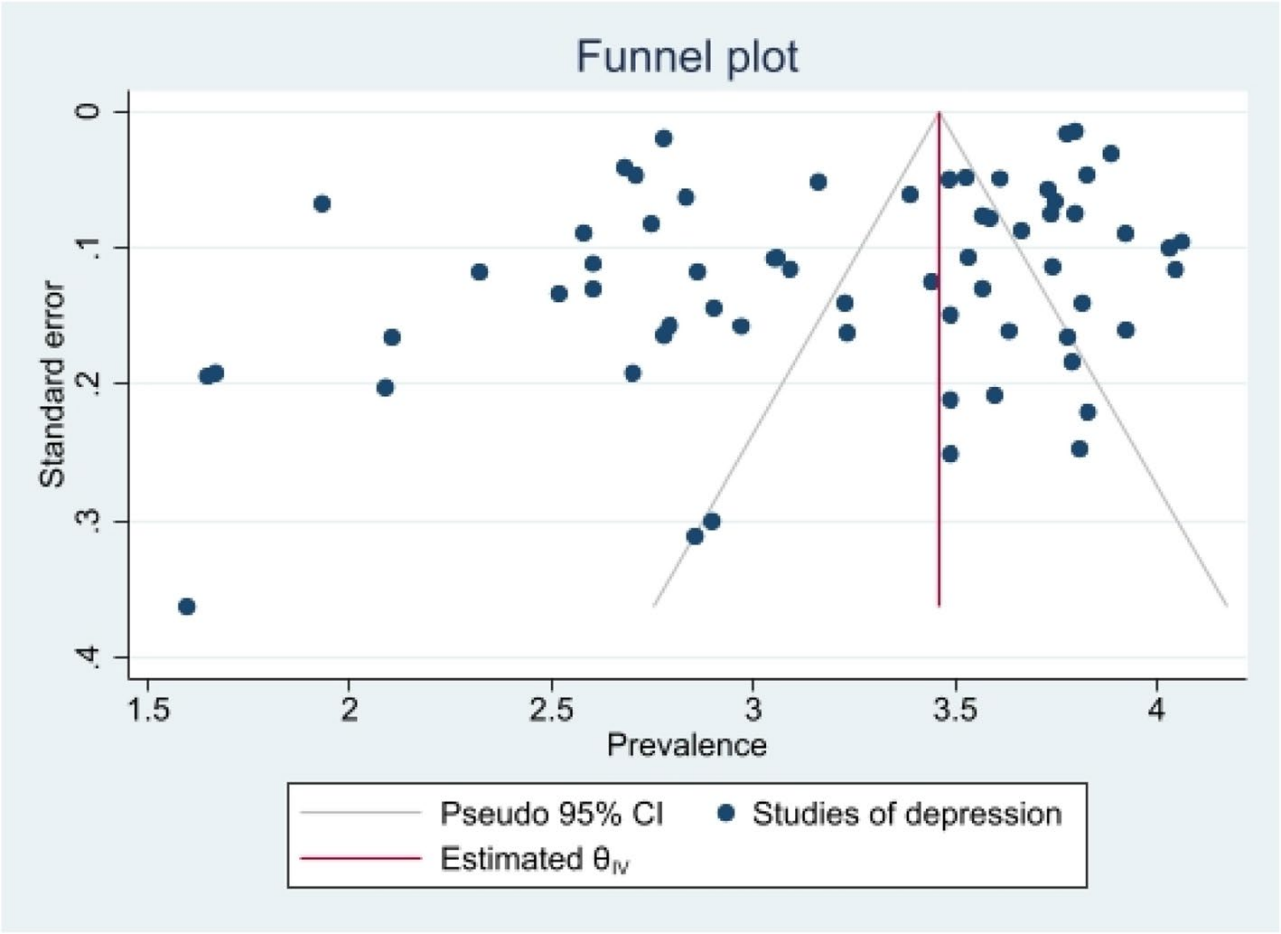
Funnel plot of depressive symptoms

**Fig. 21:**
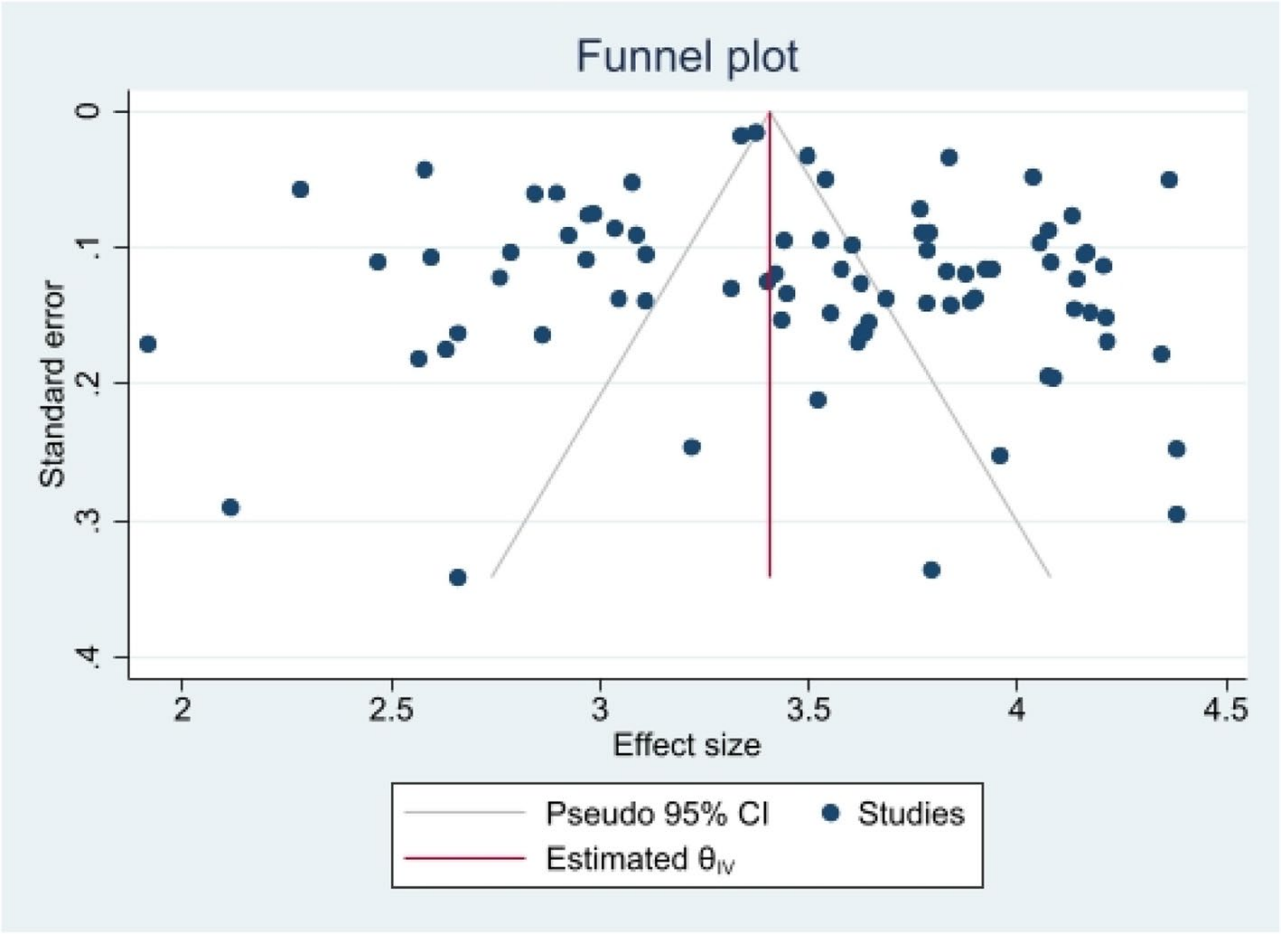
Funnel plot of anxiety symptoms

**Fig. 22:**
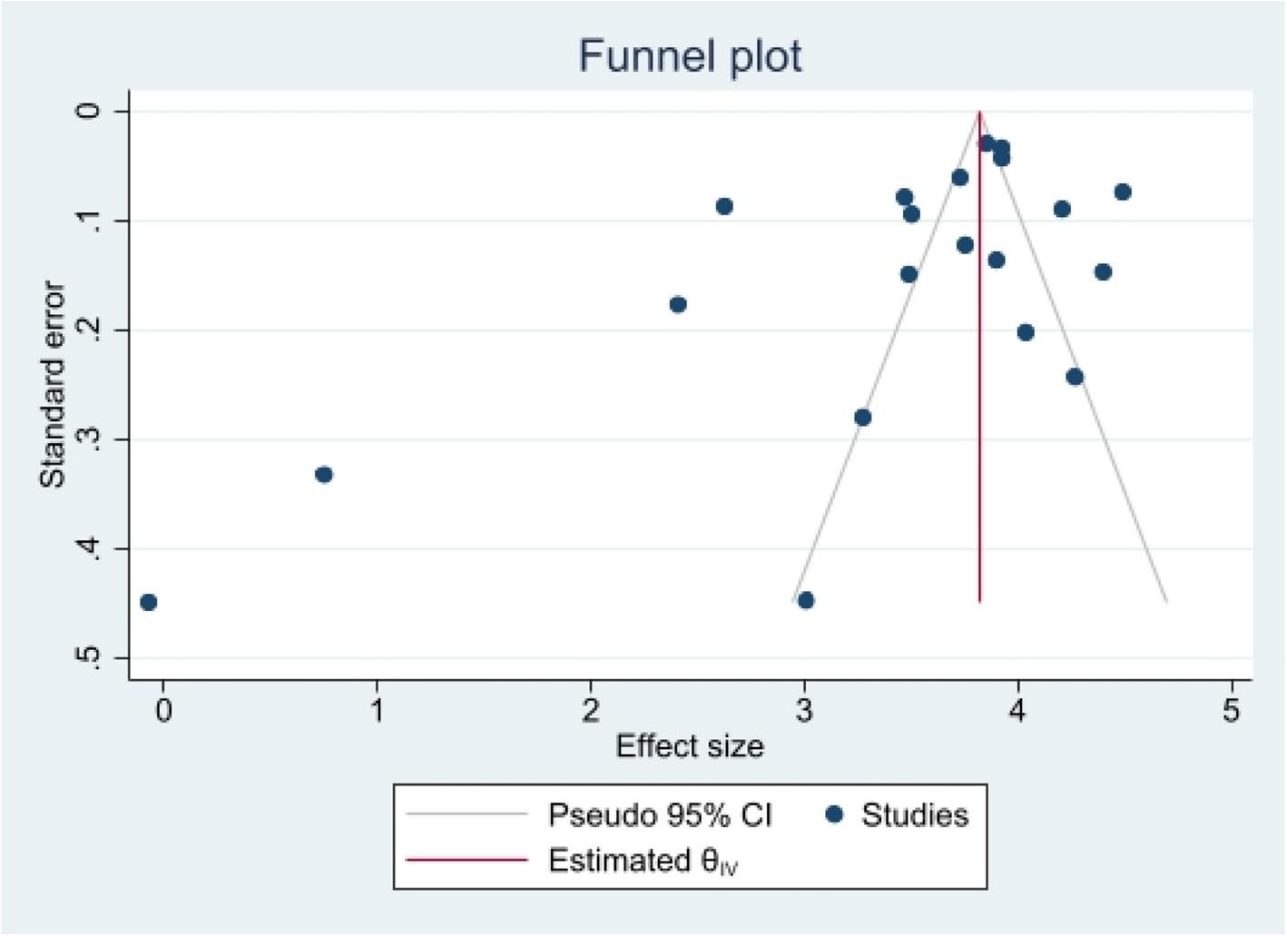
Funnel plot of stress symptoms

**Fig. 23:**
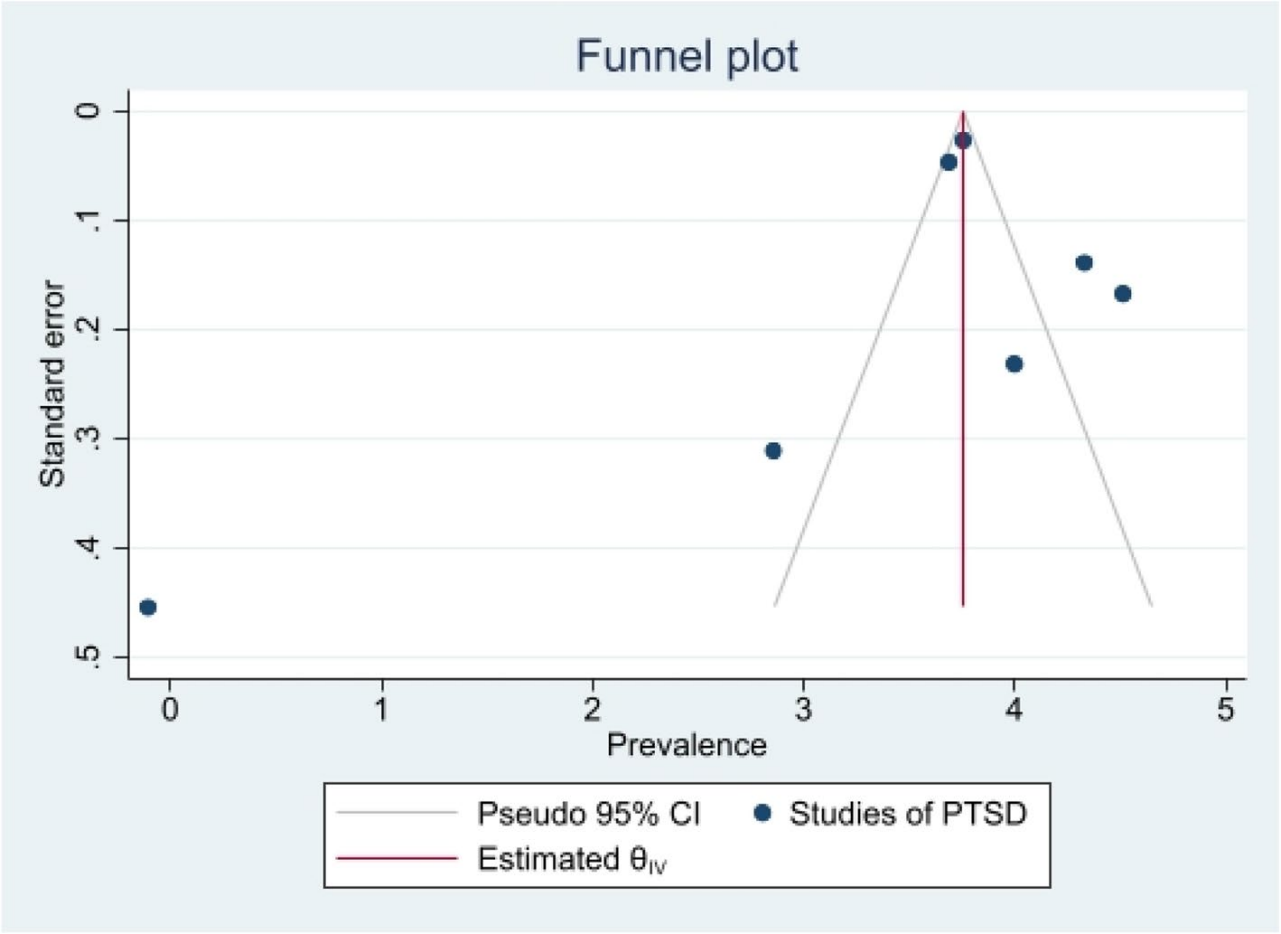
Funnel plot of PTSD symptoms

**Fig. 24:**
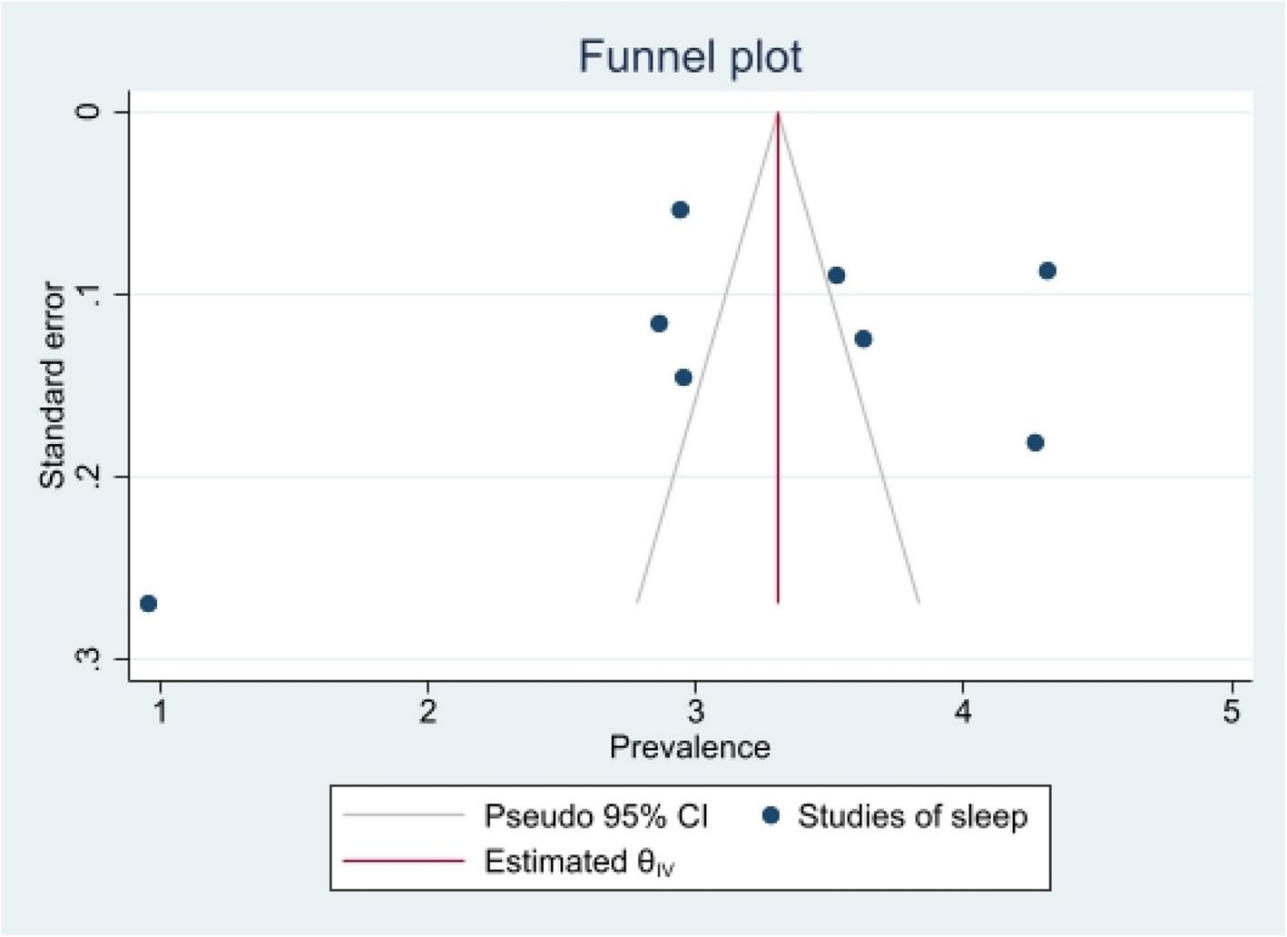
Funnel plot of sleep disorders symptoms

The heterogeneity of depressive symptoms was lower in comparison to anxiety, PTSD, stress, and sleep problems. Heterogeneity within the 1st trimester was 89.47%. I^2^ of the anxiety group during the 1st trimester and 2nd trimester were 88.91 and 92.35%, respectively. These appear to be similar to the I^2^ values of depression. I^2^ for stress associated with the 2nd and 3rd trimesters were 78.57 and 64.65%, respectively, indicating mild heterogeneity. Intuitively, Maharlouei and colleagues study reported a small prevalence, thus could be an influencing factor for the heterogeneity reported. I^2^ for PTSD across three trimesters were 24.67, 89.47 and 81.62%, respectively. I^2^ was 0% during the 1st trimester within the groups of participants reporting sleep disturbance. 1st trimester group showed relatively low heterogeneity across mental health symptoms, thus strictly stipulating the gestational weeks of the included pregnancy helped reduce the heterogeneity. Forest plots were generated for 1st trimester, 2nd trimester, 3rd trimester, post-partum and overall, for symptoms of depression (see Figs. 7, 8, 9, 10 and 11), anxiety (see Figs. 12, 13, 14, 15 and 16), stress (see Fig. 17), PTSD (see Fig. 18), sleep disorders (see Fig. 19). Funnel plots were also generated: depression (see Fig. 20), anxiety (see Fig. 21), stress, (see Fig. 22), PTSD (see Fig. 23), and sleep disorders (see Fig. 24).

#### Publication bias and sensitivity analysis

**Fig. 25:**
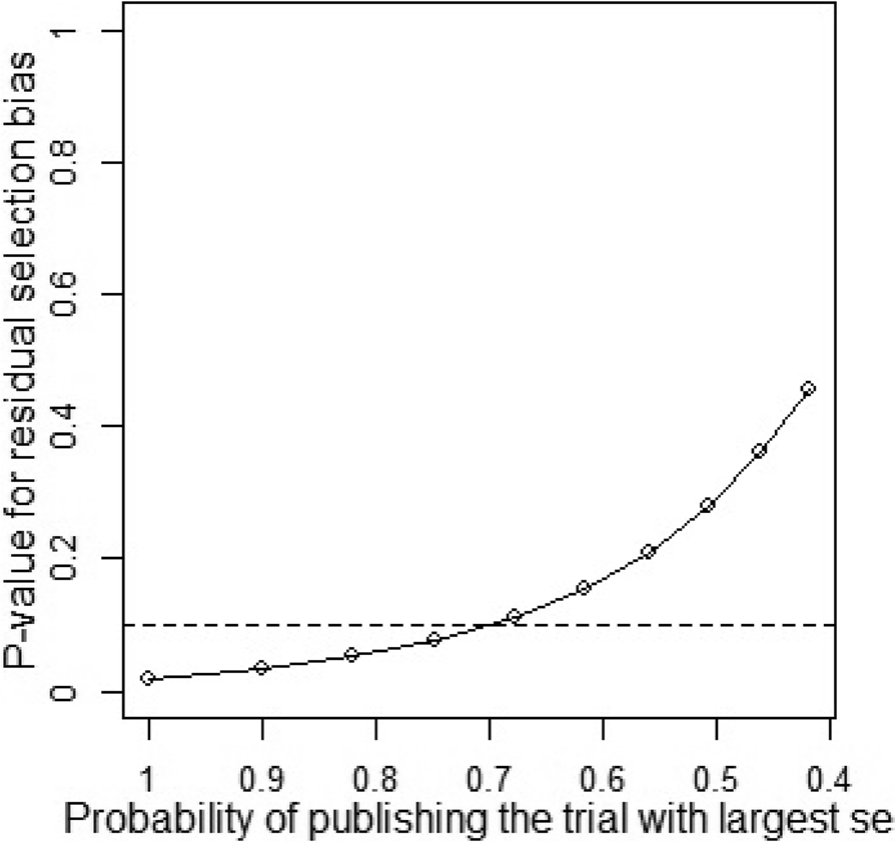
*P*-value for residual selection bias of depressive symptoms

**Fig. 26:**
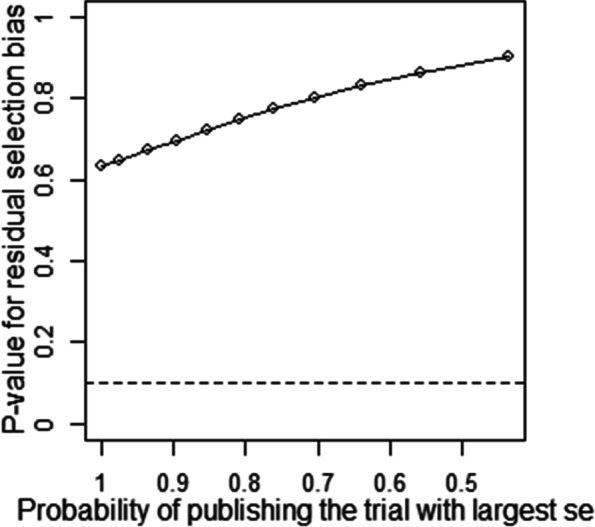
*P*-value for residual selection bias of anxiety symptoms

**Fig. 27:**
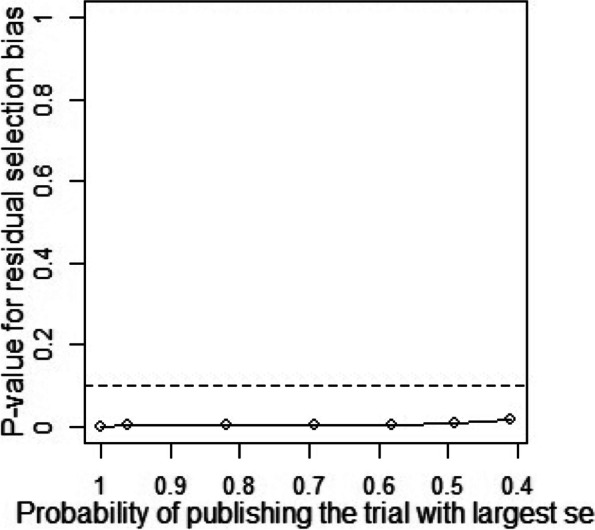
*P*-value for residual selection bias of stress symptoms

**Fig. 28:**
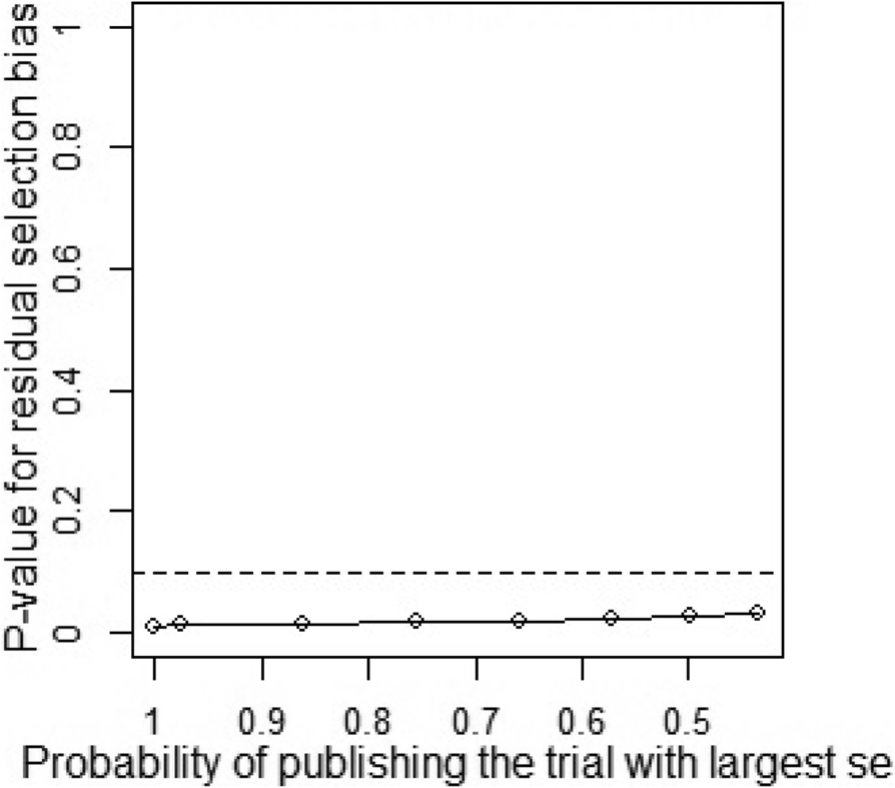
*P*-value for residual selection bias of PTSD symptoms

**Fig. 29:**
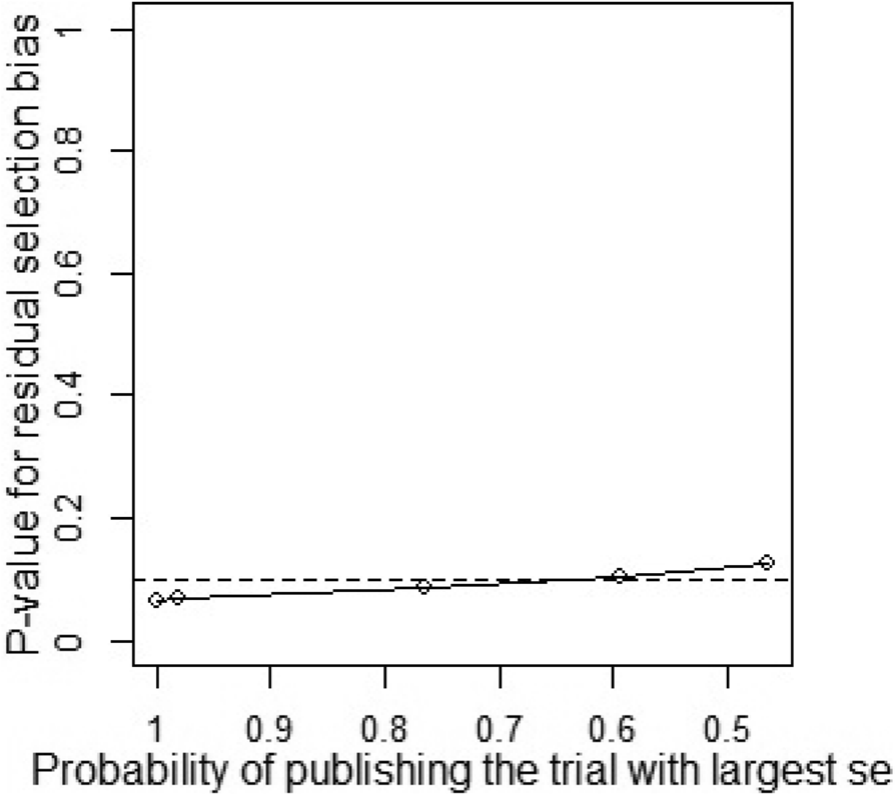
*P*-value for residual selection bias of sleep disorders symptoms

Publication bias and sensitivity analysis tests were conducted to assess the reliability of the data as some studies had large standard errors that would produce undesirable effects. Copas Selection Model was used to select studies for the sensitivity analysis. The *p*-values of residual selection bias were evaluated (see Fig. 25, 26, 27, 28 and 29). Studies with a *p*-value of > 0.1 indicated that the residual selection had minimal bias and, the selected studies can be represented. The proportions identified were 67.84, 100 and 59.49% for depression, anxiety, and sleep disorders, respectively. For studies reporting stress and PTSD, the Copas Selection Model could not provide a decision, indicating the previous conclusions of high heterogeneity is accurate.

A summary of studies used within the Copas Selection Model and Random Effects Model indicate that the two models have no significant difference (see Table 4). *P*-value of the changes between these conclusions were 0.1108 for depressive symptoms, 0.638 for anxiety symptoms, and 0.1042 for sleep disorder symptoms. The *p*-value of the Egger’s test was 0.0256 for studies of depressive symptoms, revealing the existence of publication bias (see Table 5). The *p*-values of 0.256 and 0.998 indicate that it is challenging to detect publication bias for studies associated with symptoms of anxiety and sleep disorders (see Table 5).

**Table 4:** Summary of sensitivity analysis

Outcome	N of study	Model	Probability of publishing study with largest standard error	Proportion (%)	lower (%)	upper (%)	***p-***value for differences between two conclusions
Depression	64	copas selection model	67.84%	27.11	24.32	30.22	0.1108
		random effects model		24.91	21.37	29.02	
Anxiety	82	copas selection model	100.00%	32.88	29.08	37.18	0.638
		random effects model		32.88	29.05	37.21	
Sleep disorders	8	copas selection model	59.49%	27.11	14.94	49.21	0.1042
		random effects model		24.38	11.89	49.95	

This table outlines mental health outcome, number of studies and model used to check publishing biases. Statistical analysis relevant to the publication biases have also been reported

**Table 5:** *P*-value of Egger Test for the five mental health symptoms

Outcome	N of studies	***p***-value of Egger test
Depression	64	0.0256^*^
Anxiety	82	0.256
Stress	20	0.069
PTSD	7	0.742
Sleep disorders	8	0.998

This table shows mental health outcomes, number of studies and test results for the Eggers test

Note: (*): *p* < 0.05 indicates significance

## Discussion

### Main findings

Our study demonstrates that symptoms of depression, anxiety, PTSD, stress, and sleep problems were common throughout the pregnancy period and after childbirth during the COVID-19 pandemic with 24.9% of women reporting symptoms of depression, 32.8% anxiety, 29.44% stress, 27.93% PTSD, and 24.38% sleep disorders. The lack of research conducted to assess the mental health impact of SARS and MERS on pregnant women is a significant limitation as such data could support preparation for similar pandemics in the future. Our meta-analyses indicate the clear impact of COVID-19 on the mental health of pregnant and post-partum mothers, with a pooled prevalence of the multiple symptomatology of depression, anxiety, PTSD, stress, and sleep disorder.

### Strengths and limitations

To our knowledge, this is the first systematic review and meta-analysis to focus on mental health outcomes in women during pregnancy and after childbirth during the Covid-19 pandemic. The searches were not limited by geographical location or language, therefore, further increasing the chances for all relevant literature to be identified. The MESH terms used did not consider all types of obstetric or gynaecology conditions but did include the common conditions. The variety of screening tools used across the included studies must be considered when interpreting the results of this review. Direct comparisons cannot be made where the same screening tool was not used. Furthermore, most studies used self-reported questionnaires, with no clinical follow-up to confirm diagnoses. Therefore, the results cannot be interpreted as prevalence of mental illness, but rather prevalence of symptomatology.

### Interpretation

Similar to our study, other research has demonstrated that the extent and severity of mental health impacts increased in women throughout pregnancy and after childbirth during humanitarian disasters and pandemics [224. ]. The subgroup analysis showed that the prevalence of symptoms of depression ranged from 16.52 to 24.96% across the four time points. In terms of anxiety symptoms, prevalence ranged from 22.06 to 32.09%. Likewise, Grumi et al. (2021) found prevalence of depressive and anxious symptoms ranges between 26 and 32% amongst pregnant women through the COVID-19 pandemic [225. ]. Contrary to previous findings, we found that pregnant women and women who have just given birth experience higher levels of anxiety, especially in the 1st trimester and post-partum, compared to depressive symptoms [226. ]. In terms of symptoms of anxiety and PTSD, some research has found that these symptoms have been elevated in pregnant women throughout the COVID-19 pandemic [24. ]. Women who became pregnant or gave birth during the pandemic suffered from various symptoms of poor mental health across all stages of their pregnancy and postpartum. It is unclear as to the reason for this observation, and the impact of this in a real-time scenario.

These findings could be due to pressure of being a first-time mother or, general stress and health anxiety regarding how and when to access care from midwives and obstetricians as part of routine and emergency maternity care due to the Covid-19 pandemic. Similar to our findings, other studies carried out during the Covid-19 pandemic reported up to 70% of pregnant women suffering from stress during the pandemic [8. ]. Being pregnant and giving birth are known triggers for women to develop anxiety and depression and is a known risk factor for exacerbations or decline in pre-existing mental ill-health [5. , 6. ]. Other possible reasons for the increase in mental ill-health in women during pregnancy or after childbirth may be because of the massive clinical changes that took place regarding how women could access maternity care during the Covid-19 pandemic. As pregnant women were at higher risk of severe illness if infected with SARS-CoV-2, they advised to be stringent with public health measures such as social distancing and self-isolation to lower their risk of COVID-19 exposure. This led to the rapid implementation of virtual access to antenatal care in order to minimising the need for travel to antenatal clinics and in-person contact with healthcare staff. Antenatal care changed immediately from face-to-face consultations to telephone or video consultation. Birth partners were limited in number, with visiting hours for partners restricted resulting in less emotional and psychological support for women during labour and after childbirth on the postnatal wards. Furthermore, once the Covid-19 vaccination was developed, there was uncertainty regarding the effectiveness and safety of the vaccine for pregnant women, which may also have contributed to and exacerbated stress and anxiety.

### Recommendations

All women should be risk assessed for maternal mental health at their initial visit with antenatal services and screened at every contact during pregnancy and after childbirth. All healthcare systems need to invest in perinatal mental health services, delivered from a multi-disciplinary team including mental health nurses, specialist midwives, obstetricians with specialist interest in mental health, and perinatal psychologists and psychiatrists. Maternity mental health services should be delivered in a way that meets the specific needs of the individual patient, including face-to-face consultations, telephone calls and/or video consultations. Up to date information regarding the impact of Covid-19 on maternity services needs to be available and accessible for women during pregnancy and after childbirth (e.g., through social media campaigns or hospital websites). Learning from this data, considerations of the special needs of the pregnant and postnatal mothers should be imperative in the implementation of strategies improve preparedness of the health service in future pandemics.

## Conclusion

This study highlights that maternity mental ill-health was common during the Covid-19 pandemic and highlights the need to understand the complexity of factors associated with maternal mental health. Maternity mental health services need further investment and prioritisation with clear effective referral pathways and support for women who report mental health concerns during and after pregnancy. Further research is required to explore how to best provide care in a way that meets the specific needs of each woman, across different healthcare systems.

## Data Availability

All data used within this study has been publicly available. The authors will consider sharing the dataset gathered upon request.

## Abbreviations

SARS-CoV: Severe Acute Respiratory Syndrome Coronavirus
MERS: Middle Eastern Respiratory Syndrome
SARS-Cov-2: Severe Acute Respiratory Syndrome Coronavirus 2
COVID-19: Coronavirus Disease 2019
PTSD: Post Traumatic Stress Disorder
UK: United Kingdom
PRISMA: Preferred Reporting Items for Systematic Reviews and Meta-analyses
RoB: Risk of bias
CI: Confidence interval
EPDS: Edinburgh Postnatal Depression Scale
PHQ-9: Patient Health Questionnaire 9-item
HADS-D: Hospital Anxiety and Depression Scale
STAI: State-Trait Anxiety Inventory
GAD-7: General Anxiety Disorder 7-item
SAS: Self-rating Anxiety Scale
PSS: Perceived Stress Scale
DASS21-S: 21-item Depression Anxiety and Stress Scale
PCl-5: DSM-V Post-Traumatic Stress Disorder Checklist
IES: Impact of Events Scale
ISI: Insomnia Severity Index
PSQI: Pittsburgh Sleep Quality Index
